# Epstein-Barr Virus Nuclear Antigen 3C Facilitates G1-S Transition by Stabilizing and Enhancing the Function of Cyclin D1

**DOI:** 10.1371/journal.ppat.1001275

**Published:** 2011-02-10

**Authors:** Abhik Saha, Sabyasachi Halder, Santosh K. Upadhyay, Jie Lu, Pankaj Kumar, Masanao Murakami, Qiliang Cai, Erle S. Robertson

**Affiliations:** 1 Department of Microbiology and Tumor Virology Program, Abramson Comprehensive Cancer Center, University of Pennsylvania Medical School, Philadelphia, Pennsylvania, United States of America; 2 Department of Microbiology and Infections, Kochi Medical School, Kochi University, Kochi, Japan; University of North Carolina at Chapel Hill, United States of America

## Abstract

EBNA3C, one of the Epstein-Barr virus (EBV)-encoded latent antigens, is essential for primary B-cell transformation. Cyclin D1, a key regulator of G1 to S phase progression, is tightly associated and aberrantly expressed in numerous human cancers. Previously, EBNA3C was shown to bind to Cyclin D1 *in vitro* along with Cyclin A and Cyclin E. In the present study, we provide evidence which demonstrates that EBNA3C forms a complex with Cyclin D1 in human cells. Detailed mapping experiments show that a small N-terminal region which lies between amino acids 130–160 of EBNA3C binds to two different sites of Cyclin D1- the N-terminal pRb binding domain (residues 1–50), and C-terminal domain (residues 171–240), known to regulate Cyclin D1 stability. Cyclin D1 is short-lived and ubiquitin-mediated proteasomal degradation has been targeted as a means of therapeutic intervention. Here, we show that EBNA3C stabilizes Cyclin D1 through inhibition of its poly-ubiquitination, and also increases its nuclear localization by blocking GSK3β activity. We further show that EBNA3C enhances the kinase activity of Cyclin D1/CDK6 which enables subsequent ubiquitination and degradation of pRb. EBNA3C together with Cyclin D1-CDK6 complex also efficiently nullifies the inhibitory effect of pRb on cell growth. Moreover, an sh-RNA based strategy for knock-down of both *cyclin D1* and *EBNA3C* genes in EBV transformed lymphoblastoid cell lines (LCLs) shows a significant reduction in cell-growth. Based on these results, we propose that EBNA3C can stabilize as well as enhance the functional activity of Cyclin D1 thereby facilitating the G1-S transition in EBV transformed lymphoblastoid cell lines.

## Author Summary

Epstein-Barr virus (EBV), a ubiquitous human herpesvirus, is linked to the development of multiple cancers, including lymphomas and epithelial carcinomas. EBNA3C, one of its essential latent antigens encoded by EBV, is expressed in EBV-associated lymphomas and contributes to aberrant cell growth after EBV infection. Cyclin D1 over-expression is associated with numerous cancers and is crucial for the transition from G1 to S phase in the mammalian cell-cycle. This study demonstrates that EBNA3C can enhance the functional activity of the Cyclin D1/CDK6 complex which in turn facilitates the G1 to S transition by neutralizing the growth inhibitory effects of pRb. Thus, manipulation of Cyclin D1 functions by EBNA3C provides a favorable environment to promote malignant transformation of EBV infected B-cells.

## Introduction

Epstein–Barr virus (EBV) is a B-lymphotropic human herpes virus that persists indefinitely in latently infected B-cells. EBV infection occurs early in life for most people and is associated with a broad spectrum of benign and malignant diseases including Burkitt's lymphoma (BL), nasopharyngeal carcinoma (NPC), Hodgkin's disease (HD) and lymphomas associated with immuno-compromised individuals, including AIDS patients and post-transplant patients receiving immune-suppressive therapy [1]. EBV infection in B-cell leads to aberrant cell division and under favorable conditions the infected B-cells will continue to proliferate indefinitely, resulting in development of immortalized lymphoblastoid cell lines (LCLs) [1], [2].

One of the most noteworthy EBV-host cell interactions is the establishment of viral latency. There are three major types of latency, each having its own distinct viral-gene expression pattern [1], [2]. Type I latency is usually noticed in BL tumors with predominant expression of EBV encoded nuclear antigen 1 (EBNA-1) [1], [2]. Type II latency is demonstrated in NPC and HD, where EBNA-1, latent membrane protein 1 (LMP-1), LMP-2A and -2B proteins are significantly detected [1], [2]. Type III latency, also termed as ‘growth program’ [1], [2] is typically seen in LCLs expressing six latent nuclear proteins (EBNA-1, -2, -3A, -3B, -3C, and -LP), three latent membrane proteins (LMP-1, -2A, and -2B), and the viral RNAs which includes the EBERs and BARTs (33, 62).

Molecular genetics analyses have demonstrated that at least six EBV latent genes (EBNA-1, -2, -3A, -3C, -LP, and LMP-1) are essential for *in vitro* immortalization [1], [2], indicating that a complex cascade of molecular events is required to surpass normal growth controls. One scenario which accounts for EBV-mediated B-cell immortalization is modulation of critical positive and/or negative regulators of cell-cycle progression, such as cyclins, cyclin-dependent kinases (CDKs), cyclin-dependent kinase inhibitor proteins (CDKIs), tumor-suppressors and apoptosis related proteins which includes p53 and pRb [3].

EBNA3C, one of the essential EBV latent antigens, has been shown to function both as a transcriptional activator and a repressor [4], [5], [6]. It has also been shown to interact with numerous transcription modifiers, including c-Myc [4], prothymosin α [7], histone deacetylases [8], CtBP [9], NM23-H1 [10], DP103 [11], SCF^Skp2^ [12], p300 [13] and p53 [14] which contributes to EBV induced transformation mediated by EBNA3C. In addition, a large body of evidence indicates that EBNA3C can also deregulate the cell-cycle machinery through direct protein-protein interaction and post-translational modification of important cell-cycle regulatory proteins, including Cyclin A [15], [16], pRb [17], p53 [14], Mdm2 [18], and Chk2 [19].

So far, studies probing EBNA3C functions provide perhaps the best link between latent EBV infection and the pRb regulated checkpoint which controls the G1-S phase transition [20], [21]. EBNA3C was previously shown to indirectly target pRb regulated pathways [15], [20]. EBNA3C also activates E2F-dependent promoters and can induce foci formation in colony formation assays [20]. Additionally, EBNA3C overcomes the ability of the CDK inhibitor - p16^INK4A^ to block transformation and noticeably drives serum-starved cells through the G1-S restriction point [20], [21]. More recently, we have shown that EBNA3C directly targets pRb and may indirectly target the pRb regulated checkpoint by associating with Cyclin A as well as Cyclin D1 known to be important in phophorylating pRb [15], [16]. Despite this body of evidence, a clear molecular link between these molecules responsible for disrupting the G1-S phase blockage and EBNA3C is yet to be demonstrated.

Cell-cycle progression is dependent on the activity of cyclins, a family of proteins whose levels oscillate in synchrony with cell-cycle progression, and its functional partner CDKs [22]. Cyclin D (D1, D2 and D3) is expressed in the mid-G1 phase in the mammalian cell-cycle [23]. Among the D-type cyclins, Cyclin D1 is the most ubiquitous and is frequently over-expressed in numerous human malignancies [24], [25]. Cyclin D1 over-expression is often associated with increased gene expression due to gene amplification or post-translational modification [26]. Accumulation of Cyclin D1 in cancer can result in overcoming ubiquitin-mediated degradation through several distinct mechanisms [26].

Cyclin D1, together with its catalytic partners CDK4 or CDK6, promotes G1-S-phase transition via phosphorylation of pRb and disrupting the pRb-E2F1 repressor complex [23]. These functions of Cyclin D1 ensure efficient initiation of S phase [26], [27]. During late G1 and S phases, Cyclin D1 is phosphorylated on Thr-286 by GSK3β, which triggers nuclear export and proteasomal degradation through E3 ubiquitin ligase, SCF^FBX4-αB^ ^crystallin^ [26]. Thus, subversion of either of these functions may result in unrestrained cell proliferation and oncogenesis.

The *cyclin D1* gene is located on chromosome 11q13, close to the *bcl-1* locus, and is considered to be a proto-oncogene with evidence indicating that its derangement contributes to the development of tumors [28]. Mantle cell lymphomas have been reported to over-express Cyclin D1 due to a characteristic genetic translocation [28]. In addition, patients with tumors over-expressing Cyclin D1 have been shown to have a particularly poor prognosis [25], [29]; however, over-expression of Cyclin D1 has been demonstrated for a vast series of human malignancies including breast cancers, esophageal cancers and pancreatic cancers [25], [30]. Over-expression of Cyclin D1, regardless of its gene alteration, caused abnormal cell proliferation, resulting in oncogenesis [22], [23], [31]. *cyclin D2*, considered also as a proto-oncogene, is located on chromosome 12p13, and unlike Cyclin D1, Cyclin D2 has been reported to be expressed normally in B-lymphocytes [32]. Interestingly, it has been observed that ectopic over-expression of Cyclin D2 efficiently blocks cell-cycle progression [33], suggesting an alternate role for Cyclin D2 in promoting exit from the cell-cycle and maintaining cells in a non-proliferative state. These observations suggest that D-type cyclins may have different roles depending on their levels of expression and cell type, which may also be independent of CDK activity. Reports have shown that immortalization of primary B-lymphocytes by EBV is accompanied by transcriptional activation of *cyclin D2* gene but not *cyclin D1* [32], [34]. However, Cyclin D1 protein has been shown to be significantly expressed in a number of EBV positive LCLs [35], [36] or EBV positive SCID mice lymphomas [37]. Surprisingly, these studies did not directly set out to explore the contribution of Cyclin D1 in EBV-mediated B-cell oncogenesis.

A previous study from our lab showed an *in vitro* interaction between the EBV encoded antigen EBNA3C and Cyclin D1 [16]. The experiments described in this current study explore the consequences of this interaction in terms of EBV mediated transformation of primary B-cells as well as growth maintenance of LCLs. We now show that EBNA3C stabilizes as well as enhances the kinase activity of the Cyclin D1/CDK6 complex, and the nuclear localization of Cyclin D1 to bypass the G1 restriction point. Importantly, this study provides the first evidence to show that the essential EBV latent antigen EBNA3C targets Cyclin D1, which is different from previous reports, and describes a potential fundamental mechanism by which EBV deregulates the mammalian cell-cycle in EBV-associated human cancers by facilitating the G1-S transition.

## Materials and Methods

### Plasmids, antibodies, cell lines and transfection

Myc, flag, GFP and GST tagged EBNA3C vectors have been described previously [14], [18]. pcDNA3-HA-Ub was kindly provided by George Mosialos (Aristotle University of Thessaloniki, Thessaloniki, Greece). Vectors pcDNA3-Cyclin D1, pcDNA3-1x flag-Cyclin D2 and pcDNA3-1x flag-Cyclin D3 were provided by Alan Diehl (University of Pennsylvania School of Medicine, Philadelphia) and used to generate pA3F-Cyclin D by cloning PCR amplified DNA into pA3F vector [4]. GST Cyclin D1 vectors were cloned by inserting PCR amplified DNA into pGEX-2TK vector (GE Healthcare Biosciences, Pittsburgh, PA). pGEX-Cyclin D1 (286A) was generated by PCR using pA3F-Cyclin D1 as template. Sh-RNA vector, pGIPZ (Open Biosystems, Inc. Huntsville, AL) and lentiviral packaging vectors were described [38]. CDK6 cDNA cloned into pA3F vector was derived from HEK 293 cell RNA that was purified with TRIzol reagent and reverse transcribed with Superscript II (Invitrogen, Inc., Carlsbad, CA). Mouse antibodies to Cyclin D1 (DSC-6) and Sp1 (1C6), and rabbit antibody to Ub (FL-76) were from Santa Cruz Biotechnology, Inc (Santa Cruz, CA). Rabbit antibodies to Cyclin D2 and D3 were kindly provided by Alan Diehl (University of Pennsylvania School of Medicine, Philadelphia). Mouse antibodies to flag-epitope (M2) was from Sigma-Aldrich Corp. (St. Louis, MO) and to GAPDH was from US-Biological Corp. (Swampscott, MA). Antibodies to HA-epitope (12CA5) or Myc-epitope (9E10) were prepared from cell culture supernatants as described [14], [18]. Mouse (A10) or rabbit antibody to EBNA3C were described [14], [18].

HEK 293, 293T and Saos-2 (p53^-/-^ pRb^-/-^) cells were obtained from Jon Aster (Brigham and Women's Hospital, Boston, MA, USA). Saos-2 and U2OS are human osteosarcoma cell line [39]. HEK 293, HEK 293T, U2OS, and Saos-2 cells were grown in Dulbecco's modified Eagle's medium (DMEM; HyClone, Logan, UT) supplemented with 10% fetal bovine serum (FBS; HyClone, Logan, UT), 50 U/ml penicillin (HyClone, Logan, UT), 50 µg/ml streptomycin (HyClone, Logan, UT) and 2 mM L-glutamine (HyClone, Logan, UT). BL lines BJAB, Ramos, BL41 and B95.8 infected BL41 (BL41/B95.8) were kindly provided by Elliott Kieff (Harvard Medical School, Boston, MA). MutuI, MutuIII were provided by Yan Yuan (School of Dental Medicine, University of Pennsylvania, Philadelphia, PA). These BL lines and LCL1 and LCL2 were maintained in RPMI 1640 (HyClone, Logan, UT) supplemented as described above. EBNA3C expressing BJAB lines were described [14], [18]. Unless otherwise stated all cultures were incubated at 37°C in a humidified environment supplemented with 5% CO_2_.

Adherent cells were transfected by electroporation with a Bio-Rad Gene Pulser II electroporator as described [14], [18].

### Infection of PBMCs with BAC GFP-EBV

Peripheral blood mononuclear cells (PBMC) from healthy donors were obtained from University of Pennsylvania Immunology Core. As described [40], approximately 10 million PBMC were mixed with virus supernatant in 1 ml of RPMI 1640 with 10% FBS for 4 hr at 37°C. Cells were centrifuged for 5 min at500 g, discarded the supernatant, pelleted cells and resuspended in 2 ml of complete RPMI 1640 medium in 6 well plates. EBV GFP expression visualized by fluorescence microscopy was used to quantify infection. The protein and mRNA level of the infected cells was detected after 3 days of post-infection.

### Immunoprecipitation (IP) and western blotting (WB)

Transfected cells were harvested, washed with ice cold PBS and lysed in 0.5 ml ice cold RIPA buffer [1% Nonidet P-40 (NP-40), 10 mM Tris pH 8.0, 2 mM EDTA, 150 mM NaCl, supplemented with protease inhibitors (1 mM phenylmethylsulphonyl fluoride (PMSF), 1 µg/ml each aprotinin, pepstatin and leupeptin]. Lysates were precleared with normal mouse serum plus 30 µL of Protein A/G Sepharose (1 h, 4°C). 5% of the precleared lysate was saved for input control and the protein of interest was captured by rotating the remaining lysate with 1 µg of specific antibody overnight at 4°C. Immuno-complexes were captured with 30 µl of a 1∶1 mixture of Protein-A and Protein-G Sepharose beads, pelleted and washed 5X with ice cold RIPA buffer.

For western blots, input lysates and IP complexes were boiled in laemmli buffer [41], fractionated by SDS-PAGE and transferred to a 0.45 µm nitrocellulose membrane. The membranes were then probed with specific antibodies followed by incubation with appropriate infrared-tagged secondary antibodies and viewed on an Odyssey imager. Image analysis and quantification measurements were performed using the Odyssey Infrared Imaging System application software (LiCor Inc., Lincoln, NE).

### Purification of GST fusion proteins and pull-down assays

*Escherichia coli* BL21 cells were transformed with plasmids for each glutathione S-transferase (GST) fusion protein and protein complexes containing the tagged proteins were purified essentially as described before [14], [18].

For *in vitro* binding experiments, GST fusion proteins were incubated with cell lystaes or ^35^S-labeled *in vitro*-translated protein in binding buffer (1x phosphate-buffered saline [PBS], 0.1% NP-40, 0.5 mM dithiothreitol [DTT], 10% glycerol, supplemented with protease inhibitors). *In vitro* translation was done with the TNT T7 Quick Coupled Transcription/Translation System (Promega Inc., Madison, WI) according to the manufacturer's instructions.

### Immunofluorescence

Cells were immuno-stained as described [18] with few modifications. Briefly, U2OS cells plated on coverslips were transfected with expression vectors as indicated, using Lipofectamine 2000 (Invitrogen, Carlsbad, CA) according to manufacturer's protocol. After 36 h of transfection cells were fixed. B-cells were air-dried and subsequently fixed. Transiently expressed flag-tagged Cyclin D1 was detected using M2-antibody, and GFP-EBNA3C was detected by GFP fluorescence. In B-cells, endogenously expressed Cyclin D1 and EBNA3C proteins were detected using specific antibody. The slides were examined with a Fluoview FV300 confocal microscope (Olympus Inc., Melville, NY).

### Real time quantitative PCR

Total RNA was isolated by using TRIzol reagent according to the instructions of the manufacturer (Invitrogen, Inc., Carlsbad, CA). cDNA was made by using a Superscript II reverse transcriptase kit (Invitrogen, Inc., Carlsbad, CA) according to the instructions of the manufacturer. The primers were for *cyclin D1,* 5′-TGCCCTCTGTGCCACAGATG-3′, and 5′-TCTGGAGAGGAAGCGTGTGA-3′, for *cyclin D2* 5′-TGCTCTGTGTGCCACCGACTT-3′, and 5′-CAGCTCAGTCAGGGCATCACAA-3′, for *cyclin D3* 5′-TTTGCCATGTACCCGCCATCCA-3′ and 5′-CCCGCAGGCAGTCCACTTCA-3′, and for *GAPDH* 5′-TGCACCACCAACTGCTTAG-3′ and 5′-GATGCAGGGATGATGTTC-3′. Quantitative real-time PCR analysis was done as described [18] in triplicate.

### *In vivo* poly-ubiquitination assay

15×10^6^ HEK 293T cells were transfected by electroporation with DNA vectors expressing a specific protein. Cells were incubated for 36 h and pretreated for an additional 6 h with 20 µM MG132 (Enzo Life Sciences International, Inc., Plymouth Meeting, PA) before harvesting. Proteins were immunoprecipitated with specific antibodies and resolved by SDS-PAGE. The extent of ubiquitination of immunoprecipitated complexes were detected by HA-specific antibody (12CA5) against HA-Ub tagged proteins.

### Subcellular fractionation assay

15×10^6^ HEK 293 cells were transfected with expression plasmids. After 36 h cells were PBS washed and resuspended into hypotonic buffer [5 mM Pipes (KOH) pH 8.0, 85 mM KCl, 0.5% NP-40 supplemented with protease inhibitors). After 10-min incubation on ice, cells were homogenized with 20 strokes in a Dounce homogenizer, nuclei were pelleted (2300 g for 5 min) and the cytosolic material was collected. Nuclear pellets were PBS washed, resuspended in nuclear lysis buffer (50 mM Tris, pH 8.0, 2 mM EDTA, 150 mM NaCl, 1% NP-40, and protease inhibitors), lysed by vortexing periodically for 1 h. Soluble nuclear fraction was separated by centrifugation at 21000 g for 10 min. Total protein was measured by Bradford protein assay and 50 µg of total protein was resolved by SDS-PAGE. The efficiency of nuclear and cytoplasmic fractionation was confirmed by western blot against nuclear transcription factor Sp1 and cytoplasmic protein GAPDH.

### *In vitro* kinase assay

15×10^6^ HEK 293T cells were transfected with plasmids expressing flag-Cyclin D1 (5 µg), flag-CDK6 (5 µg) and increasing amount of myc-EBNA3C (0, 5, 10, 20 µg). For GSK-3β kinase assay cells were transfected with DNA vectors that express myc-tagged GSK-3β (10 µg) and flag-tagged EBNA3C (20 µg). Cells were harvested and protein complexes were immunoprecipitated (IP) using either M2 (for cyclin D1) or 9E10 ascites fluid (for GSK-3β). IP complexes were then washed with buffer A (25 mM Tris [pH 7.5], 70 mM NaCl, 10 mM MgCl_2_, 1 mM EGTA, 1 mM DTT, plus protease and phosphatase inhibitors) and incubated in 30 µl of kinase buffer B (buffer A plus 10 mM cold ATP, and 0.2 µCi of [γ-^32^P]-ATP/µl) supplemented with either 4 µg of histone H1 (Upstate Biotechnology, Inc., Lake Placid, N.Y.) or bacterially purified GST-pRb (residues 792-928) for 30 min at 30°C. The reaction was stopped by adding 2X laemmli buffer [41] and heating to 95°C for 10 min. Labeled proteins were resolved by 12% SDS-PAGE. Band quantitation was performed using the ImageQuant software (GE Healthcare Biosciences, Pittsburgh, PA).

### Stability assay

Cells were transiently transfected using electroporation with plasmids as indicated in the text. After 36 hours transfection, cells were treated with 40 µg/ml cyclohexamide (CalBiochem, Gibbstown, NJ) and lysates were subjected to immunoblot analyses. Band intensities were quantitated using Odyssey 3.0 software provided by Odyssey imager (LiCor Inc., Lincoln, NE).

### Lentiviral shRNA expressing constructs

Short-hairpin oligonucleotides directed against EBNA3C were designed (Dharmacon Research, Chicago, IL). The sense strand of the EBNA3C-shRNA sequence is 5′-tcgagtgctgttgacagtgagcgaCCATATACCGCAAGGAATAtagtgaagccacagatgtaTATTCCTTGCGGTATATGGgtgcctactgcctcggaa-3′. The sense strand of cyclin D1 sh-RNA sequence is 5′-tcgagtgctgttgacagtgagcgaCAAACAGATCATCCGCAAAtagtgaagccacagatgtaTTTGCGGATGATCTGTTTgtgcctactgcctcggaa-3′ [42]. Upper-case letters indicate 19-nucleotide (nt) either EBNA3C or cyclin D1 target sequences respectively and lowercase letters indicate hairpin and sequences necessary for the directional cloning into pGIPZ (Open Biosystems, Inc. Huntsville, AL). Single-stranded EBNA3C and cyclin D1 oligonucleotides were first annealed and then cloned into the Xho I and Mlu I restriction sites of pGIPZ vector. The fidelity of cloned double-strand DNA was confirmed by DNA sequencing. In parallel, a commonly available control shRNA sequence (Dharmacon Research, Chicago, IL): (5′-TCTCGCTTGGGCGAGAGTAAG-3′) that lacks complementary sequences in the human genome was also cloned into pGIPZ vector.

### Lentivirus production and transduction of EBV-transformed B-cells

Lentivirus production and transduction of EBV-transformed B-cells (LCLs) were essentially carried out as previously described [38].

### Proliferation assay

Saos-2 (p53^-/-^ pRb^-/-^) were transfected using Ca_3_(PO4)_2_ method as described [38]. After 24 h transfection, cells were selected using DMEM supplemented with 1000 µg/ml G418; Invitrogen). After a 2-week selection, 5×10^6^ cells were harvested, lysed in RIPA buffer and subjected for immunoblot analyses. Approximately 0.1×10^6^ cells from each set of samples were plated into each well of the 6-well plates and cultured for 6 days. Viable cells from each well were counted by trypan blue exclusion method daily using a Bio-Rad TC10 Automated cell counter.

For LCLs, approximately 1×10^6^ cells were plated into each well of the 6-well plates and cultured at 37°C in complete RPMI medium. Cells were counted similarly for 20 days. Both experiments were performed in duplicate and were repeated two times.

### Colony formation assay

5×10^6^ Saos-2 (pRb^-/-^) cells were transfected as described [38] and cultured in DMEM supplemented with 1 mg/ml G418 (Invitrogen, Inc., Carlsbad, CA). After a 2-week selection, cells were fixed on the plates with 4% formaldehyde and stained with 0.1% crystal violet (Sigma-Aldrich Corp., St. Louis, MO). The area of the colonies (pixels) in each dish was calculated by Image J software (Adobe Inc., San Jose, CA). The data are shown as the average of three independent experiments.

### Cell-cycle analysis

For serum starvation experiments, the culture medium was replaced with RPMI 1640 and 0.1% FBS for 12 h. Cells were PBS washed, fixed in cold 70% ethanol for 30 min at 20°C, PBS washed and stained 2 h in buffer containing 50 mg/ml propidium iodide, 10 mM Tris pH 7.5, and 500 U/ml RNAseA in dark. PBS washed cells were analyzed for cell-cycle profile by FACS Calibur system and Cellquest software (Becton-Dickinson Inc., San Jose, CA).

## Results

### EBV infection leads to induction of Cyclin D1

**Figure 1:**
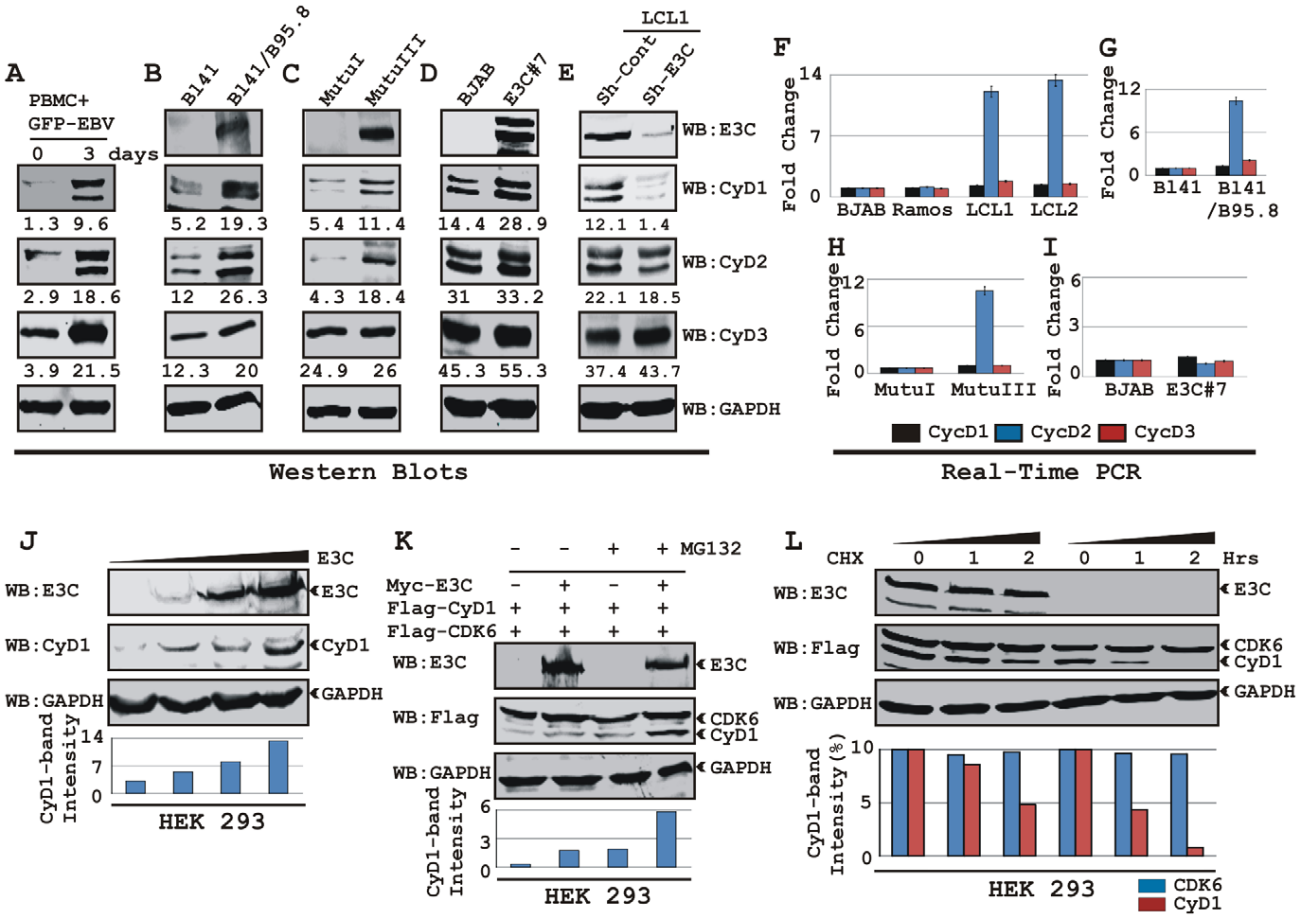
EBV nuclear antigen EBNA3C stabilizes Cyclin D1 protein level. A) 10 million human peripheral blood mononuclear cells (PBMC) were infected by BAC GFP-EBV for 4 h. At 3-days post-infection cells were lysed in RIPA buffer and western blots of endogenous proteins were probed with the indicated antibodies. B-E) 20 million cells of (B) Burkitt's lymphoma (BL) cell line BL41 and BL41 cells infected with wild-type EBV strain B95.8 (BL41/B95.8); (C) type I and III latency BL cell lines - MutuI cells (latency I gene expression program) and MutuIII cells (latency III gene expression program); (D) BJAB cells and BJAB cells stably expressing EBNA3C (BJAB_E3C#7); (E) *EBNA3C* and *cyclin D1* knock-down LCL1 cells (LCL1_Sh-E3C and LCL1_Sh-CyD1 respectively) were harvested and total cell proteins were subjected to Western blot (WB) analysis using indicated antibodies. F-I) Total RNA was isolated from cells F) BJAB, Ramos, LCL1 and LCL2; G) BL41 and BL41/B95.8; H) MutuI and MutuIII; I) BJAB and BJAB E3C3 7 and were individually subjected to quantitative real-time PCR analysis for detecting *cyclin D1*, *D2 and D3* transcript levels. Each sample was tested in triplicate and data obtained from three independent experiments were expressed as the difference of the quantity of specific transcripts to the quantity of GAPDH transcript as control. The fold change in expression of each *cyclin D* mRNA relative to GAPDH was calculated based on the threshold cycle (Ct) as 2^- Δ(ΔCt)^, where ΔCt  =  Ct_target_ – Ct_GAPDH_ and Δ(ΔCt)  =  ΔCt_test sample_ - ΔCt_control sample_. J) HEK 293 cells were transfected with an increasing amount (0, 2, 5, 20 µg) of EBNA3C expressing construct and western blot analysis was performed to detect EBNA3C, Cyclin D1 and GAPDH. K) HEK 293 cells were co-transfected with flag-Cyclin D1 and either vector control (lanes 1 and 3) or myc-EBNA3C (lanes 2 and 4). At 36 h posttransfection, samples were treated with either 40 µM MG132 (+ lanes) or DMSO (- lanes) for 6 h and resolved by 10% SDS-PAGE and probed with the indicated antibodies. L) HEK 293 cells were similarly transfected with expression plasmids for flag-tagged both Cyclin D1 and CDK6 and myc-tagged EBNA3C as indicated. At 36 h post-transfection, cells were treated with 40 µg/ml cyclohexamide (CHX) for indicated lengths of time. 10% of the lysate from each sample were resolved by 10% SDS-PAGE. GAPDH blot was done for loading control. Western blotting was done by stripping and reprobing the same membrane. Protein bands were quantified using Odyssey imager software as indicated either as arbitrary numerical values at the bottom of gel (A-E) or as bar diagrams (J-L) based on GAPDH loading control.

In order to determine whether EBV infection alters Cyclin D expression, approximately 10×10^6^ human resting peripheral blood mononuclear cells (PBMC) were infected by BAC GFP-EBV as previously described [40] for 4 h and western blot analysis was performed on samples collected 3 days after infection. The results showed that EBV infection leads to a significant induction of all three Cyclin D protein levels 3 days post-infection, with no preference for any particular D-type cyclins (Fig. 1A). Similarly, western blot results of Burkitt's lymphoma (BL) cell line BL41 and BL41 infected with wild-type EBV strain B95.8 (BL41/B95.8) also showed elevated levels of Cyclin Ds with Cyclin D1 expression more dramatically changed compared to other Cyclin Ds (Fig. 1B). Since Cyclin D1 expression was induced significantly after EBV infection in both PBMC and BL cell line, we next wanted to determine if the induction was related to a specific EBV latent protein expressed during type III latency. The results showed that the levels of both Cyclin D1 and Cyclin D2 proteins were induced in type III latency BL cell line MutuIII compared to latency I expressing MutuI BL cell line (Fig. 1C). These results differ with previously published observations which suggested that B-cells infected with EBV do not express Cyclin D1 [43], [44], [45]. However, in agreement with previously published results [32], our real-time PCR data showed that EBV infection led to a significant increase of *cyclin D2* mRNA level in LCLs (LCL1 and LCL2) when compared to EBV negative BL cells (BJAB and Ramos) whereas, there was little or no detectable change for *cyclin D1* mRNA (Fig. 1F). Real-time PCR data obtained from two other matched sets of cell lines BL41 – BL41/B95.8 and MutuI – MutuIII also showed similar results as above (Fig. 1G and 1H, respectively). These results suggest that D-type cyclins are regulated through distinctly different mechanisms in EBV infected B-cells. EBV effects on Cyclin D2 are at the level of its transcript stability whereas the effects on Cyclin D1 or D3 seem to be post-translational.

### EBNA3C expression leads to stabilization of Cyclin D1

To elucidate the effects of the EBV encoded essential nuclear antigen, EBNA3C on Cyclin D1, BL lines BJAB and E3C #7, a BJAB stably expressing EBNA3C were analyzed. The western blot results showed a significant increase in Cyclin D1 protein expression among D-type Cyclins in E3C #7 cells compared to the BJAB control cells and smaller changes in Cyclin D2 and D3 (Fig. 1D). The effect of EBNA3C on Cyclin D1 steady-state levels was not due to changes in the transcription as EBNA3C expression did not alter the level of *cyclin D1* mRNAs in these cells as seen above (Fig. 1I). To further verify the role of EBNA3C on Cyclin D1 protein accumulation, we determined the levels of Cyclin Ds in a lymphoblastoid cell line with the EBNA3C mRNA specifically targeted by short-hairpin RNA (Sh-E3C). The western blot data showed that the expression level of Cyclin D1 in the LCLs stably knocked-down for EBNA3C (Sh-E3C) was significantly diminished as compared to the control cell line (Sh-Control) (Fig. 1E), however the expression levels of other Cyclin Ds was not altered (Fig. 1E). These results indicate that EBNA3C can contribute to Cyclin D1 accumulation in latently infected EBV positive cells.

To demonstrate that EBNA3C can stabilize Cyclin D1 protein levels, HEK 293 cells were transfected with an increasing amount of an expression construct expressing EBNA3C and tested for endogenous Cyclin D1 protein level. The results showed that EBNA3C stabilizes Cyclin D1 protein expression in a dose dependent manner (Fig. 1J).

We earlier determined that EBNA3C plays a critical role in modulating the ubiquitin (Ub)-proteasome machinery [12], [17], [18]. Therefore, to investigate whether the increase of Cyclin D1 levels was because of the inhibition of Ub-proteasome mediated destabilization by EBNA3C, transiently co-transfected cells were treated with the proteasome inhibitor, MG132. The results showed that both the treatment with MG132, and presence of EBNA3C led to a significant accumulation (six fold) of Cyclin D1 when compared to mock treatment or vector control (Fig. 1K). Therefore the increased levels of Cyclin D1 observed in the presence of EBNA3C and MG132 is a result of stabilization of Cyclin D1 likely by EBNA3C inhibition of the Ub-proteasome degradation system. Importantly, both CDK6 and EBNA3C levels were not altered by MG132 (Fig. 1K).

To directly determine EBNA3C stabilization of Cyclin D1, HEK 293 cells were transfected with flag-Cyclin D1, flag-CDK6, and EBNA3C expression vectors. Thirty-six hours later, cells were treated with protein synthesis inhibitor cycloheximide, and samples were collected at 0, 1, and 2 hours. Western blots probed with flag antibody showed that the stability of Cyclin D1 protein was significantly enhanced by EBNA3C co-expression, whereas in the absence of EBNA3C, Cyclin D1 was degraded to near completion by 2-h after addition of CHX (Fig. 1L, grey bar). Cyclin D1 half life was determined to be 2 h in EBNA3C expressing cells; however, it shortened noticeably to less than 1 h when Cyclin D1 was expressed alone (Fig. 1L, bar diagram). The results also indicated that both EBNA3C and CDK6 were notably stable throughout the experimental period of time and had no sign of protein degradation (Fig. 1L, CDK6 indicated as black bar). Overall, the results of these experiments suggest EBNA3C can stabilize Cyclin D1 by regulating its targeted degradation likely through the Ub-proteasome degradation system.

### EBNA3C stabilizes Cyclin D1 via inhibiting its poly-ubiquitination

**Figure 2:**
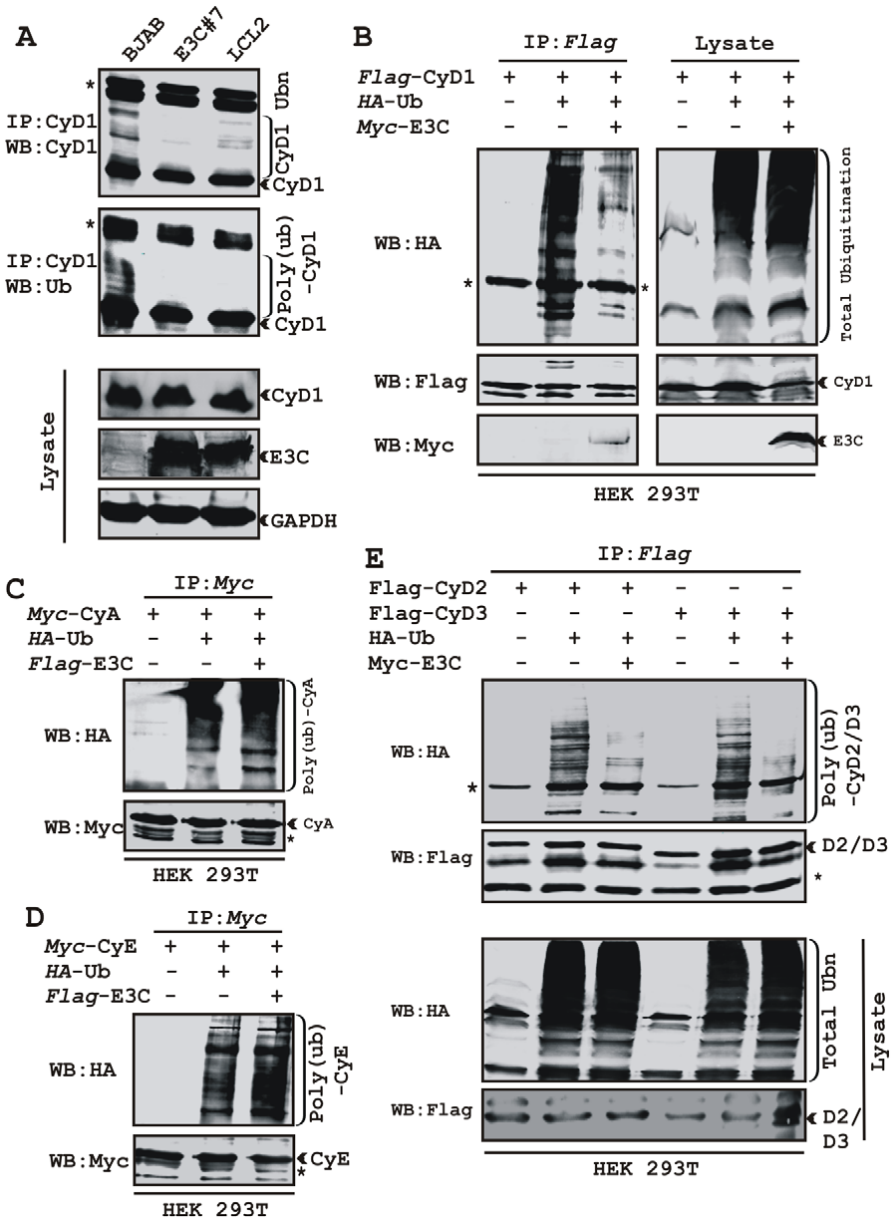
EBNA3C stabilizes Cyclin D1 through inhibiting its poly-ubiquitination. A) 50 million EBV negative BJAB cells, BJAB cells stably expressing EBNA3C (BJAB_E3C#7) and an EBV transformed cell, LCL2 were harvested after 6h incubation with proteasome inhibitor MG132 (20 µM). Cells were lysed and Cyclin D1 was immunoprecipitated (IP). Samples were resolved by 10% SDS-PAGE. Western blotting (WB) was done by stripping and reprobing the same membrane. B-E) 15 million HEK 293T cells were transiently transfected with different combinations of expression plasmids as indicated. Cells were harvested at 36h, and total protein was immunoprecipitated (IP) with indicated antibody and samples were resolved by 10% SDS-PAGE. Western blotting was done by stripping and reprobing the same membrane. Asterisks (*) indicate the immunoglobulin bands and poly-(ub) indicates poly-ubiquitination.

Recently we have shown that ectopic expression of EBNA3C leads to stabilization of an important cellular oncoprotein, Mdm2 by inhibiting its poly-ubiquitination [18]. The increased stability of Cyclin D1 in the presence of EBNA3C, prompted us to examine whether EBNA3C similarly inhibits poly-ubiquitination of Cyclin D1 and so enhances its stability. To explore this possibility, three cell lines were selected, the EBV negative cell line BJAB, BJAB stably expressing EBNA3C (E3C #7) and an EBV positive lymphoblastoid cell line (LCL2). Immnuprecipitation using specific antibody against Cyclin D1 resulted in formation of high molecular weight species of Cyclin D1 migrating at a slower rate in BJAB cells while in BJAB cells stably expressing EBNA3C or in LCL2 significantly less of these high molecular weight bands were observed (Fig. 2A). Re-probing of the same membrane with Ub specific antibody showed a similar pattern (Fig. 2A). This result indicates that the activity responsible for the change in Cyclin D1 bands is present in EBV positive cells (LCL2) and EBNA3C expressing cell line (E3C #7) when compared to the EBV negative BJAB cells.

To directly address this phenomenon, an ubiquitination experiment was set up, where HEK 293T cells were transiently co-transfected with expression constructs for HA-Ub, flag-Cyclin D1 and myc-EBNA3C and the ubiquitination of the Cyclin D1 was assessed by immunoprecipitation followed by Western blotting (Fig. 2B). The result demonstrated a significant and reproducible reduction in Cyclin D1 poly-ubiquitination level in EBNA3C expressing cells (Fig. 2B). Similar experiments were performed separately using two different cyclins, Cyclin A and Cyclin E to determine if this effect was specific for Cyclin D1. However, neither Cyclin A nor Cyclin E poly-ubiquitination levels were reduced in the presence of EBNA3C (Fig. 2C and 2D). To determine whether the poly-ubiquitination level of the other D-type cyclins was also affected in the presence of EBNA3C, we tested flag-tagged Cyclin D2 and D3 for ubiquitination in the absence and presence of EBNA3C. Importantly, poly-ubiquitination of both Cyclin D2 and D3 was efficiently inhibited in the presence of EBNA3C (Fig. 2E). This result indicates that EBNA3C can profoundly affect the poly-ubiquitination of all Cyclin Ds and thus enhance their stability.

### EBNA3C forms a complex with Cyclin D1 in human cells

**Figure 3:**
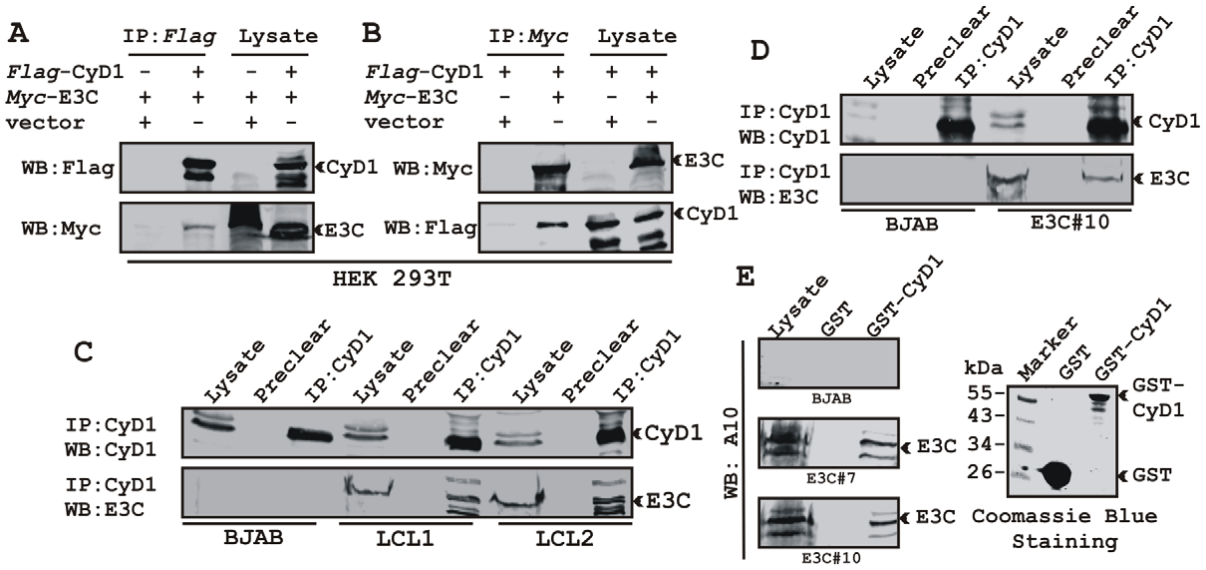
EBNA3C forms a complex with Cyclin D1 in human cells. A-B) 15 million HEK 293T cells were co-transfected with myc-tagged EBNA3C and flag-tagged Cyclin D1 vectors. In each case control samples were balanced with empty vector. Cells were harvested at 36 h post-transfection and approximately 5% of the lysed cells were saved as input and the residual lysate was immunoprecipitated (IP) with 1 µg of indicated antibody. Lysates and IP complexes were resolved by 10% SDS-PAGE and western blotted (WB) with the indicated antibodies. C) 50 million BJAB cells and two different clones of EBV transformed lymphoblastoid cell lines - LCL1 and LCL2, and in D) BJAB cells stably expressing EBNA3C (BJAB_E3C#10) along with BJAB control cells were collected and lysed in RIPA buffer. Protein complexes were immunoprecipitated with Cyclin D1 specific antibody and samples were resolved by a 10% SDS-PAGE followed by western blot with antibodies as indicated. E) Either GST control or GST-cyclin D1 beads were incubated with lysates prepared from 50 million BJAB cells and two different clones of BJAB cells stably expressing EBNA3C (BJAB_E3C#7 and #10). EBNA3C was detected by western blot with the specific monoclonal antibody (A10). Coomassie staining of a 12% SDS-PAGE resolved purified GST and GST-Cyclin D1 proteins used in this study is shown in the right panel.

We have shown earlier that EBNA3C interacts with Cyclin D1 *in vitro* along with other cyclins including Cyclin A and Cyclin E [16]. In order to determine whether EBNA3C forms a complex with Cyclin D1 in cells to enhance its stability, we performed binding assays using co-IP experiments. HEK 293T cells were co-transfected with expression constructs for myc*-*EBNA3C and flag-Cyclin D1. The results showed that ectopically expressed EBNA3C associated with Cyclin D1 in cells (Fig. 3A and 3B). To further determine whether this binding occurred under endogenous settings, Cyclin D1 was immunoprecipitated from EBV negative cell line, BJAB and two EBV transformed lymphoblastoid cell lines, LCL1 and LCL2 expressing EBNA3C. EBNA3C was detected by Western blot analysis using A10, an EBNA3C specific monoclonal antibody and showed efficient co-immunoprecipitation (Fig. 3C). In a separate experimental setting, Cyclin D1 was immunoprecipitated from BJAB cells and BJAB cells stably expressing EBNA3C (E3C#10). Similarly co-IP of EBNA3C was demonstrated using the A10 antibody (Fig. 3D). To further corroborate the association in human cells, a GST-pulldown experiment was conducted; where bacterially expressed GST-Cyclin D1 was incubated with cell lysates prepared from either BJAB cells or BJAB cells stably expressing EBNA3C (E3C#7 and E3C#10). EBNA3C was seen to strongly associate with GST-Cyclin D1 but not with the GST control (Fig. 3E). Coomassie staining of a parallel gel showed the amount of GST and GST-Cyclin D1 proteins used in the binding assay (Fig. 3E, right panel). Analysis of the data from the ectopic expression system as well as cell lines endogenously expressing Cyclin D1 and EBNA3C at physiological levels strongly demonstrated an association between Cyclin D1 and EBNA3C in human cells.

### A small N-terminal region of EBNA3C binds to two different sites of Cyclin D1

**Figure 4:**
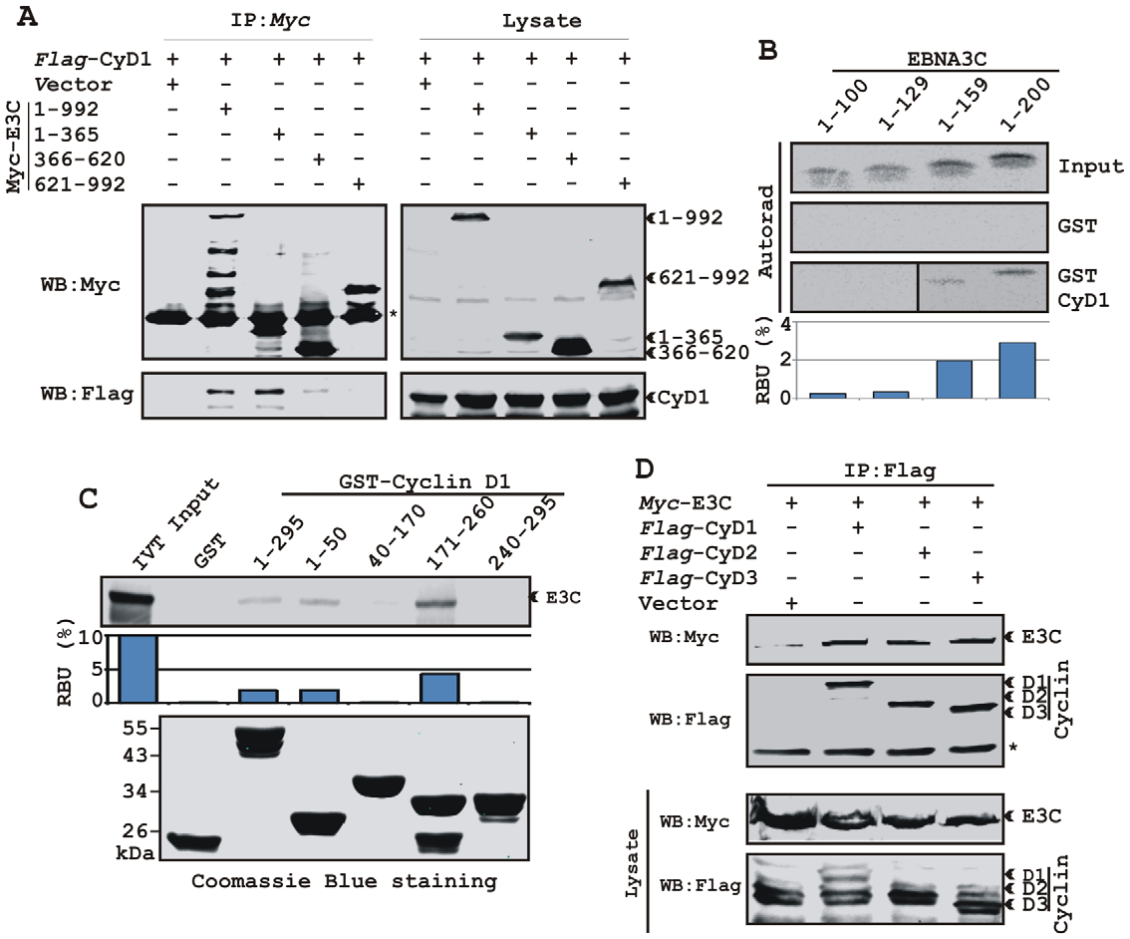
A small N-terminal region of EBNA3C binds to two different sites of Cyclin D1. A) 15 million HEK 293T cells were co-transfected with vectors expressing flag-tagged Cyclin D1 and myc-tagged EBNA3C including full-length EBNA3C (residues 1-992) or different truncated mutants (residues 1–365, 366–620 and 621–992), as indicated. Cells were harvested at 36 h and 5% of the lysed cells were saved as input and the residual lysate was immunoprecipitated (IP) with 1 µg anti-myc antibody. Samples were resolved by 10% SDS-PAGE and transferred to 0.45 µm nitrocellulose membrane. The membrane was probed with flag antibody to detect co-immunoprecipitated Cyclin D1. The membrane was stripped and reprobed with anti-myc antibody to check the IP efficiency. B) Different truncated mutant constructs of EBNA3C (residues 1–100, 1–129, 1–159 and 1–200) were *in vitro* translated using a T7-TNT translation kit. All S^35^-radiolabeled *in vitro* translated proteins in binding buffer were precleared by rotating with GST-beads for 1 h at 4°C. Binding reactions were setup by incubating the *in vitro* translated proteins with either GST control or GST-Cyclin D1 overnight. Reaction samples were resolved by 15% SDS-PAGE, exposed to phosphoimager plate and scanned on a Storm 850 imaging system. C) A series of N- and C-terminal deletion mutants of GST-fused Cyclin D1 protein were purified and tested for their ability to bind *in vitro* translated S^35^-radiolabeled full-length EBNA3C as similar to (B). Coomassie staining of SDS-PAGE resolved purified GST proteins is shown in the bottom panel of (C). D) 15 million HEK 293T cells were co-transfected with myc-tagged EBNA3C and either flag-tagged Cyclin D1, Cyclin D2 or Cyclin D3. Cells were harvested at 36 h post-transfection and subjected to immunoprecipitation with 1 ug myc antibody. Lysates and IP complexes were resolved by 10% SDS-PAGE and western blotted (WB) with the indicated antibodies. Asterisks (*) indicate the immunoglobulin bands.

We have previously shown that a small N-terminal region of EBNA3C (residues 130-160) binds to Cyclin D1 *in vitro* [16]. To map the domain of EBNA3C that interacts with Cyclin D1, HEK 293T cells were transfected with expression constructs for flag*-*Cyclin D1 and either full-length EBNA3C (residues 1-992), EBNA3C residues 1-365, EBNA3C residues 366–620, or EBNA3C residues 621-992. All EBNA3C expression constructs were fused in frame with a myc epitope tag at the C-terminus of the protein. As expected, the results showed that Cyclin D1 co-immunoprecipitated with full-length EBNA3C as well as with the N-terminal domain of EBNA3C (residues 1–365) (Fig 4A, left-middle panel, lanes 2 and 3, respectively) whereas no co-IP was detected with vector control or other truncated versions of EBNA3C (Fig 4A, left-middle panel, lanes 1, 4 and 5). To further corroborate the binding data, an *in vitro* GST-pulldown experiment was performed using *in vitro* translated^ 35^S-radiolabeled fragments of EBNA3C (residues 1–100, 1–129, 1–159 and 1–200) within the N-terminal domain. *In vitro* precipitation experiments with bacterially expressed GST-Cyclin D1 showed strong association with residues 1–159 and 1-200 of EBNA3C (Fig. 4B, bottom panel, lanes 3 and 4, respectively), but not with EBNA3C residues 1–100 or 1–129 (Fig. 4B, bottom panel, lanes 1 and 2, respectively). All fragments of EBNA3C failed to interact with the GST control, indicating that the observed binding was specific for Cyclin D1 (Fig. 4B, middle panel, lanes 1 to 4).

In an attempt to gain insights into the functionality of the association between Cyclin D1 and EBNA3C, a series of N- and C-terminal deletion mutants of Cyclin D1 (residues 1–50, 40–170, 171–260 and 241–295) were designed according to their domain distribution [46], [47] and tested for their ability to bind EBNA3C using *in vitro* binding experiments. The results of the GST-pulldown assay clearly showed that full-length Cyclin D1, the N-terminal pRb binding region (residues 1-50) and the C-terminal domain which is known to regulate Cyclin D1 stability (residues 171–260) strongly associated with EBNA3C (Fig. 4C, top panel, lanes 3, 4 and 6, respectively). However, no binding was detected with the other truncated versions of Cyclin D1 (the CDK4/6 binding domain, residues 40–170 and the PEST domain, residues 241–295) or with the GST control (Fig. 4C, top panel, lanes 2, 5, and 7). Importantly, the C-terminal domain of Cyclin D1 (residues 171–260) bound to EBNA3C with relatively higher affinity than the full-length or the N-terminal site (Fig 4C).

In order to determine the specificity of EBNA3C and Cyclin D1 interaction, we next performed a co-immunoprecipitation assay using all three flag-tagged D-type Cyclins. Interestingly, the results showed that EBNA3C forms complexes with all three D-type Cyclins in cells, suggesting that EBNA3C has specificity for interaction with Cyclin D1, D2 and D3 (Fig. 4D).

### EBNA3C can promote nuclear localization of Cyclin D1

**Figure 5:**
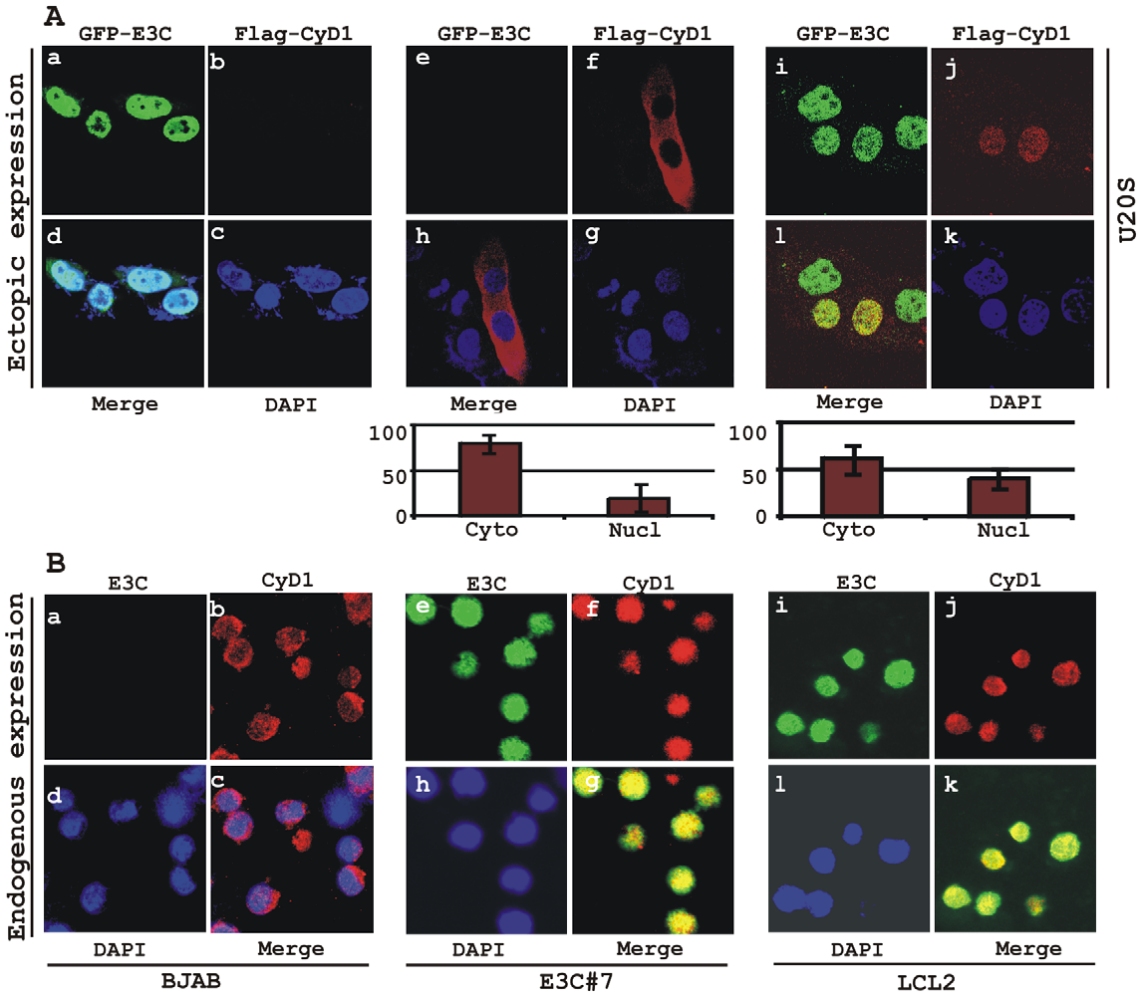
EBNA3C expression leads to an increase in nuclear retention of Cyclin D1. A) U2OS cells plated on coverslips and transiently transfected with GFP-EBNA3C and flag-Cyclin D1 using Lipofectamine 2000. B) BJAB, BJAB cells stably expressing EBNA3C (BJAB_E3C#7) and an EBV transformed lymphoblastoid cell line, LCL1 were plated and air-dried onto slides. Cells were fixed using a 1∶1 mixture of acetone and methanol. Ectopically and endogenously expressed Cyclin D1 was detected using M2-antibody (1∶200 dilution) and DCS-6 (1∶50 dilution) respectively, followed by anti-mouse Alexa Fluor 594 (red). Endogenous EBNA3C in stable cell line and in EBV positive cells was detected using an EBNA3C-reactive rabbit serum (1∶150 dilution) followed by anti-rabbit Alexa Fluor 488 (green). The nuclei were counterstained using DAPI (4′,6′-diamidino-2-phenylindole). The images were sequentially captured using an Olympus confocal microscope. In (A) the bar diagram represents the mean value of 10 different fields of three independent experiments of Cyclin D1 cytoplasmic and nuclear localization.

Increased expression of Cyclin D1 has been seen in a number of cancers [25], [30]; however, this enhanced expression is usually not sufficient to drive the oncogenic process. Emerging evidence suggests that nuclear accumulation of Cyclin D1 resulting from altered nuclear trafficking and proteolysis is critical for its oncogenic phenotype [31]. In order to determine the effect of EBNA3C on the sub-cellular localization of Cyclin D1, asynchronously growing U2OS cells were transfected with expression vectors encoding flag-tagged Cyclin D1 and GFP-tagged EBNA3C. Localization of Cyclin D1 was determined by indirect immunofluorescence using a monoclonal antibody against the flag epitope (Fig. 5A, panels f, h, j, l). While Cyclin D1 mostly localized to the cytoplasm in the absence of EBNA3C (Fig. 5A, panels f, h), it was predominantly localized to the nucleus in the presence of EBNA3C (Fig. 5A, panels j, l). To quantitatively compare the Cyclin D1 signals in the nuclear and cytoplasmic compartments, 10 different fields of the stained slides were examined and the bar diagram represents the mean of three independent experiments which showed that nuclear localization was increased by 20% (Fig. 5A, bar diagram). To further corroborate these results showing that EBNA3C promotes nuclear localization of Cyclin D1, the sub-cellular localization of endogenous Cyclin D1 was determined in three different cell lines – EBV negative BL cell line BJAB, BJAB cells stably expressing EBNA3C (E3C# 7) and an EBV transformed B-cell line LCL2, using a specific antibody against cyclin D1. As anticipated, the results showed that cyclin D1 was predominantly localized in the nucleus of both EBNA3C positive BJAB cells (Fig. 5B, panels f, g) and EBV positive cells LCL2 (Fig. 5B, panels j, k), but was almost exclusively cytoplasmic in the EBV negative BJAB cells with no EBNA3C expressed (Fig. 5B, panels b, c).

**Figure 6:**
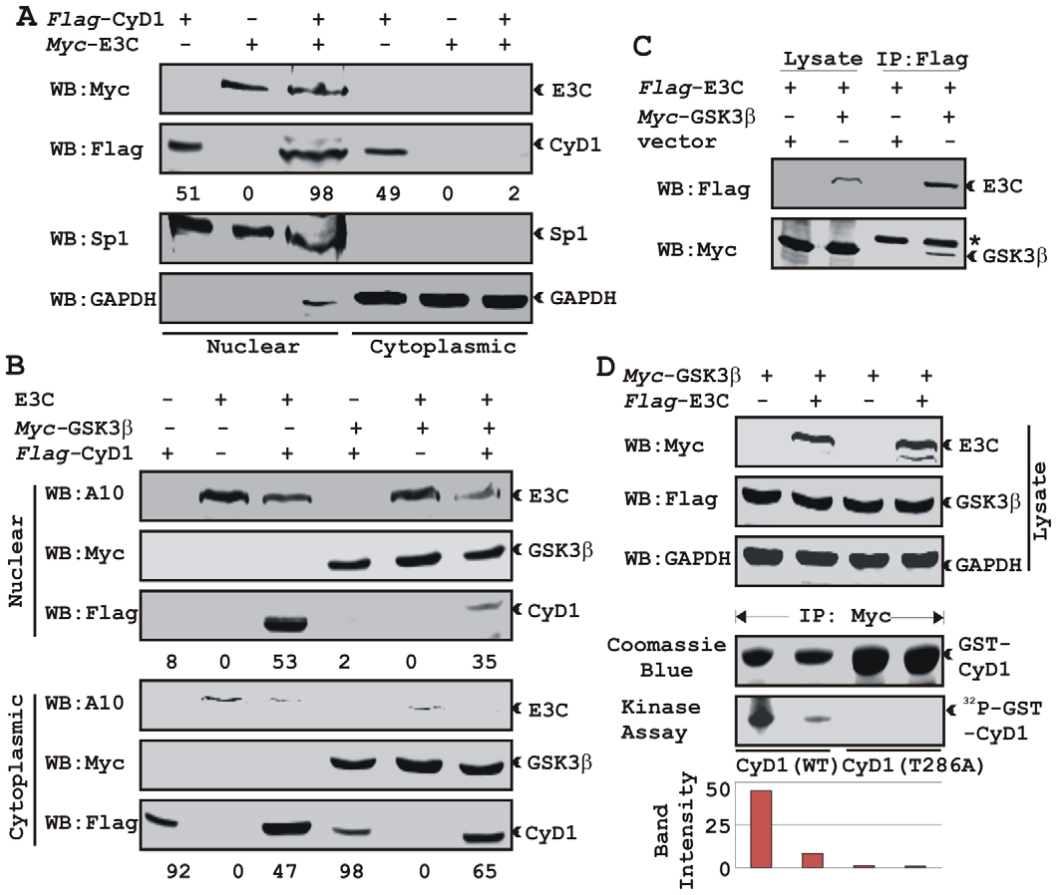
EBNA3C bypasses GSK3β dependent nuclear export of Cyclin D1. A) 15 million HEK 293 cells were transiently co-transfected with vectors of flag-tagged Cyclin D1 and myc-tagged EBNA3C and subjected to sub-cellular fractionation. Fractionated proteins were analyzed by probing western blots with flag and myc antibodies. Nuclear protein Sp1 and cytoplasmic protein GAPDH were immuno-detected as control. B) As similar to (A) 15 million HEK 293 cells were transfected with Cyclin D1 and different combinations of expression constructs as indicated and subjected to sub-cellular fractionation. The fractionated proteins were analyzed by using indicated antibodies. The percent distribution of Cyclin D1 is shown as numbers below panel (A) and (B). C) 15 million HEK 293T cells were co-transfected with myc-tagged GSK-3β and flag-tagged EBNA3C expression vectors. Cells proteins were collected 36 h post-transfection and immunoprecipitated (IP) with 1 ug of flag antibody. Lysates and IP complexes were resolved by 10% SDS-PAGE and western blotted (WB) with the indicated antibodies. Asterisk indicates the immunoglobulin bands. D) HEK 293T cells were transfected with myc-tagged GSK-3β and flag-tagged EBNA3C vectors as indicated. Empty vector was used to balance total transfected DNA. At 36 h posttransfction, GSK-3β immunoprecipitates were captured with myc antibody and assayed for *in vitro* kinase activity toward either recombinant GST-Cyclin D1 (lanes 1 and 2) or GST-Cyclin D1 T286 mutant (lanes 3 and 4) using γP^32^-ATP. Western blot using whole cell lysates are shown in first three panels and coomassie staining of SDS-PAGE resolved recombinant GST proteins used in this study is shown in panel 4.

Based on immuno-fluorescence studies, we observed that Cyclin D1 localization was mainly restricted to the cytoplasmic fraction of asynchronously growing cells. However, on expression of EBNA3C the localization of Cyclin D1 was predominantly nuclear. To further support these data, transiently transfected HEK 293 cells were subjected to sub-cellular fractionation and fractionated proteins were analyzed by immunoblot analysis. The result showed that flag-tagged Cyclin D1 alone was detected approximately 50% in both cytoplasmic and nuclear fractions, respectively (Fig. 6A, compare lanes 1 and 4). However, when co-transfected with EBNA3C, flag-Cyclin D1 was detected predominantly within the nuclear fraction (Fig. 6A, compare lanes 3 and 6), with an approximately 50% increase compared to flag-Cyclin D1 alone (Fig. 6A, compare lanes 1 and 3). EBNA3C was detected completely within nuclear fraction (Fig. 6A, lanes 2 and 3). The efficiency of cytoplasmic and nuclear fractionation was confirmed by localization of nuclear transcription factor Sp1 and cytoplasmic protein GAPDH (Fig. 6A). These observations strongly suggested that the apparent nuclear trans-localization of Cyclin D1 mediated by EBNA3C, as determined by indirect immuno-fluorescence microscopy or sub-cellular fractionation assay may be due to deregulation of the critical regulatory kinase GSK-3β, a negative regulator of Cyclin D1 nuclear retention and protein stability [31]. We thus decided to examine whether EBNA3C can nullify the effect of GSK-3β on Cyclin D1 function.

### EBNA3C blocks GSK3β dependent nuclear export of Cyclin D1

GSK-3β can direct the nuclear export of Cyclin D1 via a CRM1-dependent pathway [31]. To examine whether EBNA3C can block Cyclin D1 nuclear export, we tested the ability of EBNA3C to override GSK-3β triggered Cyclin D1 nuclear export. To test this possibility, HEK 293 cells were transfected with expression vectors encoding flag-tagged Cyclin D1, with or without GSK-3β and myc-tagged EBNA3C. Fractionated cell lysates were analyzed by western blot to clarify flag-tagged Cyclin D1 localization. As expected, Cyclin D1 was primarily present in the cytoplasmic fraction both in the absence and presence of GSK-3β (Fig. 6B, lanes 1 and 4). In contrast, Cyclin D1 was largely detected within the nuclear fraction when co-expressed with EBNA3C (Fig. 6B, lane 3). Interestingly, even in the presence of GSK-3β nuclear fractionation of Cyclin D1 was greatly increased when co-expressed with EBNA3C compared with the vector control (Fig. 6B, compare lanes 1 and 3).

GSK-3β has been shown to phosphorylate Cyclin D1 on Thr-286 *in vitro* [31], and is postulated to be a major regulator of protein levels and intracellular distribution of Cyclin D1 [31]. To establish a plausible explanation for the inhibitory effects of EBNA3C on GSK-3β dependent Cyclin D1 subcellular localization, we first asked whether EBNA3C can form a complex with GSK-3β to negatively modulate its activity and to also determine whether the kinase activity of GSK-3β is inhibited in the presence of EBNA3C. To this end, we co-expressed myc-tagged GSK-3β and flag-tagged EBNA3C and assessed their interaction through co-immunoprecipitation experiment. The results showed that indeed EBNA3C can form a complex with GSK-3β (Fig. 6C, compare lanes 3 and 4). Next, an *in vitro* kinase assay was conducted where GSK-3β was immuno-precipitated in the absence and presence of EBNA3C, and tested for its ability to phosphorylate recombinant GST-Cyclin D1 proteins (wild-type and T286A mutant Cyclin D1). The results showed that the phosphorylation level of wild-type GST-Cyclin D1 by GSK-3β was reduced by more than 4 fold in the presence of EBNA3C (Fig. 6D, compare lanes 1 and 2). As expected, no phosphorylation bands were observed in case of mutant GST-Cyclin D1 (T286A) indicating the specificity of this experiment (Fig. 6D, lanes 3 and 4). Parallel blots showed the protein expression levels in whole cell-lysate (Fig. 6D), and the amount of purified GST-Cyclin D1 used in this experiment (Fig. 6D). These results indicated that EBNA3C may regulate Cyclin D1 sub-cellular localization probably by blocking the function of GSK-3β.

### EBNA3C enhances Cyclin D1-dependent kinase activity

**Figure 7:**
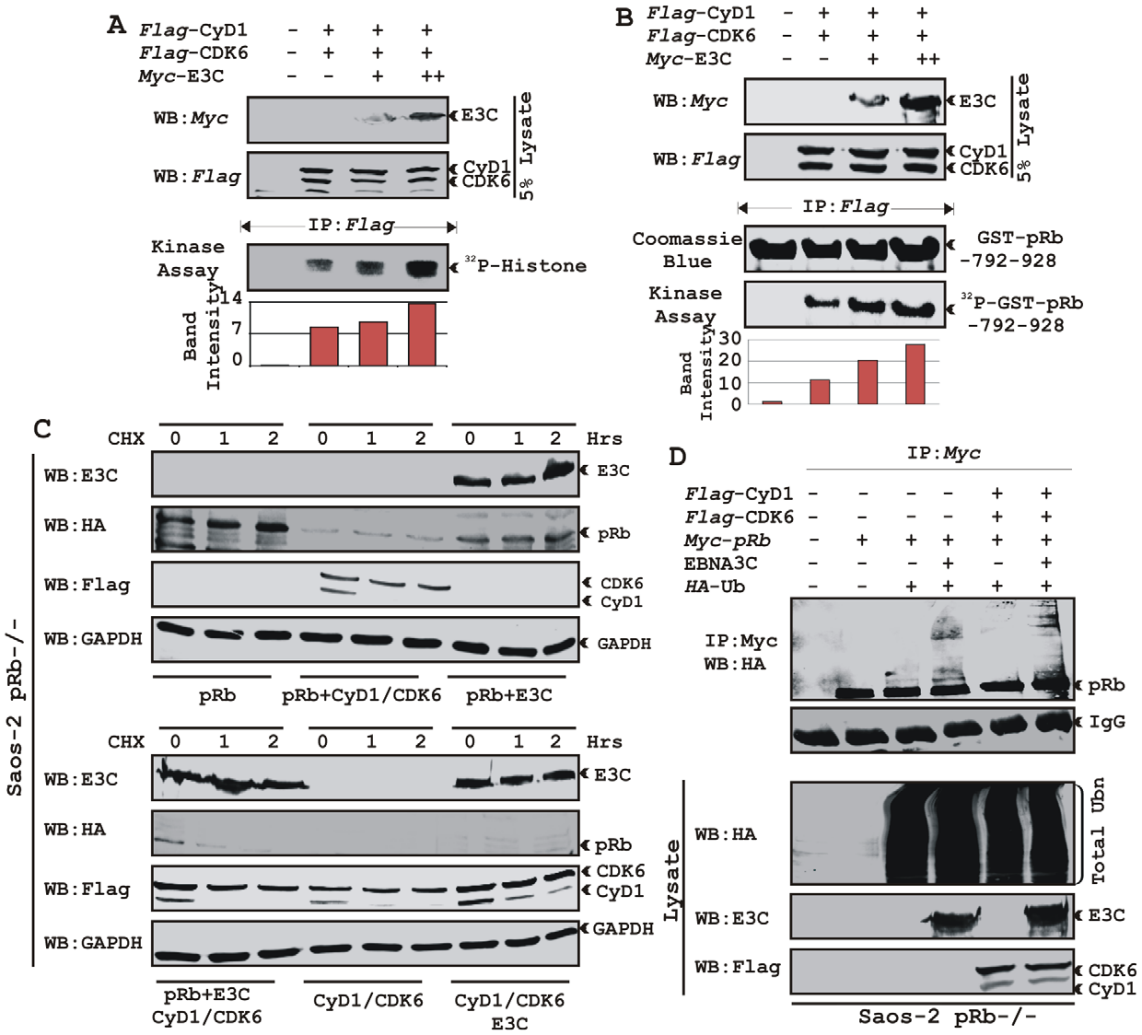
EBNA3C enhances functional activity of Cyclin D1/CDK6 complex to negatively regulate pRb protein stability. A–B) Analysis of Cyclin D1/CDK6 mediated phosphorylation of Histone H1 and pRb. HEK 293T cells were transfected with flag-tagged Cyclin D1 and CDK6 vectors and 0, 5, or 15 µg of myc-tagged EBNA3C vector. 36 h post-transfection, flag immunoprecipitates were captured and assayed for *in vitro* kinase activity on (A) Histone H1 or (B) recombinant GST-pRb (residues 792-928) as similar to figure 6D. C) Stability assay of pRb. Saos-2 (pRb^-/-^) cells were co-transfected with expression plasmids for myc- tagged pRb, flag-tagged Cyclin D1 and CDK6, and EBNA3C. 36 h post-transfection, cells were treated with 40 µg/ml cycloheximide (CHX) for the indicated times. Samples were resolved by SDS-PAGE. GAPDH was immunodetected to normalize protein levels. Western blots were probed with the indicated antibodies. D) Ubiquitination of pRb. HEK 293T cells were transfected with expression plasmids for myc-tagged pRb, HA-tagged ubiquitin (Ub), and EBNA3C (E3C), and flag-tagged Cyclin D1/CDK6 as indicated. Cells were harvested at 36 h, and total protein was immunoprecipitated (IP) with myc-specific antibody. Samples were resolved by SDS-PAGE. Western blots were probed with the indicated antibodies.

To address the functional consequences as a result of the association of Cyclin D1 and EBNA3C, we tested the activity of Cyclin D1/CDK6 complexes for the ability to phosphorylate histone H1 or recombinant GST-pRb (residues 792-928). HEK 293T cells were transiently transfected with increasing amounts of a myc-tagged EBNA3C expression construct. Flag-tagged Cyclin D1/CDK6 immunoprecipitated complexes were assayed for *in vitro* kinase activity as determined by histone H1 or GST-pRb phosphorylation (Fig. 7A and B, respectively). The results showed that Cyclin D1-dependent kinase activity increased in a dose-responsive manner with increased expression of EBNA3C (Fig. 7A and B). Phosphorimager analysis revealed 1.6-times more P^32^-Histone H1 and 2.3-times more P^32^-GST-pRb (Fig. 7A and B). Parallel blots showed the expressed protein levels (Fig. 7A and B, top two panels) and the amount of substrates (histone H1 or GST-pRb) used in this study (Fig. 7A and B).

### EBNA3C can reduce the half-life of pRb by regulating the kinase activity of the cyclin D1/CDK6 complex

Cyclin D1/CDK4/6 complexes are rate-limiting for G1 progression by contributing to the sequential phosphorylation of pRb, and thereby canceling the growth-suppressive function of pRb, thus facilitating entry into S-phase [26], [27]. Previously we have shown that EBNA3C facilitates pRb degradation by enhancing its poly-ubiquitination through recruitment of the SCF^Skp2^ E3 ligase activity [17]. To test whether EBNA3C coupled with Cyclin D1/CDK6 complex regulates pRb stabilization, a stability assay was performed using cycloheximide (CHX) treated Saos-2 (pRb^-/-^ p53^-/-^) cells co-transfected with plasmids expressing myc-tagged pRb, flag-tagged Cyclin D1, flag-tagged CDK6, and EBNA3C (Fig. 7C). The results clearly showed that independent expression of either Cyclin D1/CDK6 complex or EBNA3C reduced pRb expression levels (Fig. 7C [upper panel], compare lanes 1-9). Surprisingly, when both EBNA3C and Cyclin D1/CDK6 complex were expressed together, little or no pRb was detected (Fig. 7C [lower panel], lanes 1-3), indicating that EBNA3C can also facilitate pRb degradation in cooperation with Cyclin D1/CDK6 either through stabilization of Cyclin D1 (Fig. 7C [lower panel], compare lanes 4–9) or increasing kinase activity of Cyclin D1/CDK6 complex.

### EBNA3C can enhance pRb ubiquitination in a Cyclin D1-dependent manner

In order to probe whether EBNA3C enhances pRb poly-ubiquitination in a Cyclin D1-dependent manner for degradation, we performed an *in vivo* ubiquitination assay. HEK 293T cells were co-transfected with expression constructs for myc-tagged pRb, *HA*-tagged Ub, flag-tagged Cyclin D1 and CDK6 and untagged EBNA3C as indicated (Fig. 7D). pRb was immunoprecipitated with myc antibody, and ubiquitinated-pRb was detected by probing blots with HA antibody. In agreement with the previous result, poly-ubiquitination of pRb was significantly enhanced in the presence of EBNA3C alone (Fig. 7D, compare lanes 3 and 4) and slightly further elevated in the presence of both EBNA3C and Cyclin D1/CDK6 complex (Fig. 7D, compare lanes 4 and 6) indicating that EBNA3C together with Cyclin D1/CDK6 is important for inducing pRb poly-ubiquitination and its subsequent degradation.

### EBNA3C nullifies the negative regulation of cell proliferation by pRb

**Figure 8:**
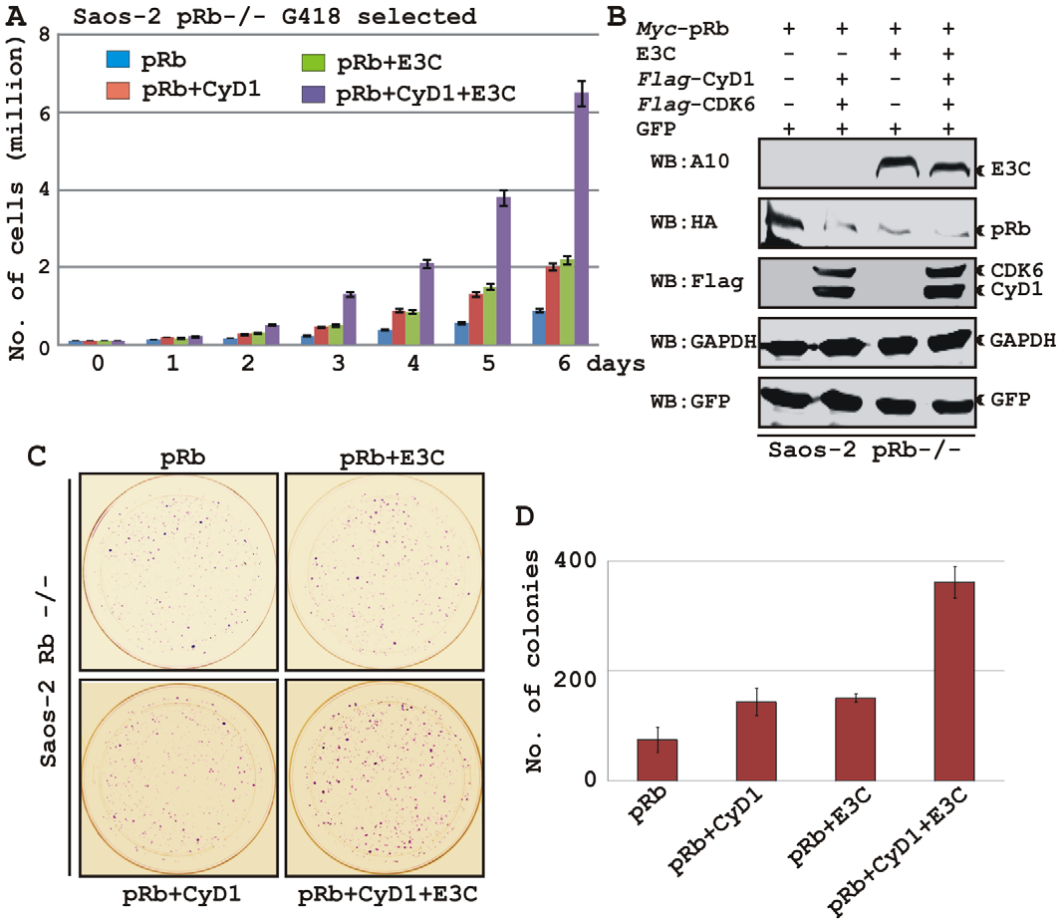
EBNA3C coupled with Cyclin D1/CDK6 complex nullifies the growth suppressive effect by pRb. A-B) Saos-2 cells were transfected with expression plasmids for myc-tagged pRb, flag-tagged Cyclin D1 and CDK6 and EBNA3C. Cells were additionally transfected with GFP expression vector. Cells were selected for 2 weeks with G418. A) Approximately 0.1×10^6^ cells from each set of samples were plated into each well of the 6-well plates and cultured for 6 days. Viable cells from each well were counted by trypan blue exclusion method daily using an automated cell counter. For (B) 5×10^6^ cells were harvested, lysed in RIPA buffer and subjected for immunoblot analyses using indicated antibodies. C–D) Saos-2 cells transfected with different combinations of expression plasmids as described in panel (A) and selected similarly as stated above with G418. After a 2-week selection, cells were fixed on the plates with 4% formaldehyde and stained with 0.1% crystal violet. The area of stained cells in each dish was calculated by Image J software. A and D) The bar diagram represents the average data of two independent experiments with standard deviation.

To determine the effect of EBNA3C and Cyclin D1/CDK6 complex on pRb mediated cell growth suppression, an osteosarcoma cell line, Saos2, was transfected with the expression plasmids for myc-tagged pRb, flag-tagged Cyclin D1, flag-tagged CDK6 and EBNA3C as indicated in the figure (Fig. 8A–D). Cells were additionally transfected with a GFP expression vector. The cells were selected with G418 for 2 weeks and the proliferation rate of the selected cells was measured by an automated cell counter for 6 days (Fig. 8). Dead cells were excluded using Trypan Blue staining. The rationale for choosing Saos2 as recipient cells was that cell growth suppression and morphological changes can be observed in Saos2 cells that express pRb de novo [48]. The results showed that EBNA3C together with Cyclin D1/CDK6 complex effectively reduced the growth suppressive effect of pRb. The cell-proliferation rate of cells expressing pRb either with EBNA3C or Cyclin D1/CDK6 complex was 1.5-2 fold higher than pRb alone (Fig. 8A). However, interestingly EBNA3C together with Cyclin D1/CDK6 complex significantly enhanced the cell proliferation rate, which was approximately either 6 fold higher than pRb alone or 3 fold higher than pRb when co-expressed with either EBNA3C or Cyclin D1/CDK6 complex (Fig. 8A). To check the expression levels of these proteins, the selected cells were subjected to western blot analysis (Fig. 8B). The results showed that the pRb expression levels were significantly reduced in EBNA3C or Cyclin D1/CDK6 expressing samples, whereas no changes of expression were observed for other proteins (Fig. 8B). GAPDH was used as an internal loading control and expression of GFP indicated equivalent amount of total protein lysate prepared from selected cells (Fig. 8B).

In order to corroborate the previous experiment, we next performed a colony formation assay, where cells were similarly transfected with different combinations of expression constructs as stated above. After selection of the transfected cells with G418 similarly as stated above for 2 weeks, the number of antibiotic-resistant colonies was counted (Fig. 8C–D). In agreement with the previous experiment, the results showed that co-expression of both EBNA3C and Cyclin D1/CDK6 proteins with pRb in Saos-2 cells resulted in an increase in the number of colonies compared to pRb alone (Fig. 8C, compare panels 1–3 and Fig. 8D, bar diagram). However, interestingly EBNA3C together with Cyclin D1/CDK6 complex markedly increased the antibiotic-resistant colonies (Fig. 8C, compare panels 1-4 and Fig. 8D, bar diagram). Overall, these results indicate that EBNA3C can utilize the function of Cyclin D1/CDK6 to neutralize the growth inhibitory effect of pRb.

### EBNA3C and Cyclin D1 are required for cell-cycle progression in EBV transformed cells

**Figure 9:**
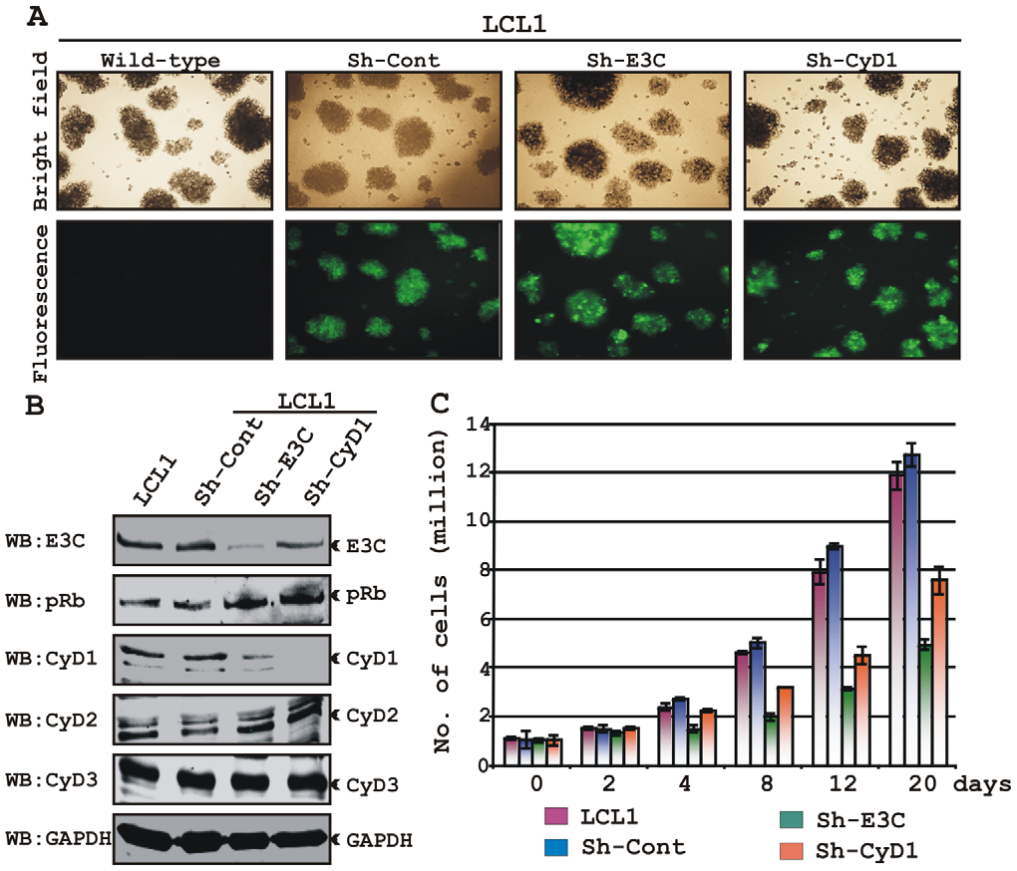
Both EBNA3C and Cyclin D1 are required for cell-cycle progression in EBV transformed cells. (A) Lentivirus transduced short hairpin RNA vectors knock down EBNA3C and Cyclin D1 in EBV transformed LCLs. Transduction with sh-RNA-containing lentivirus and selection of EBV-infected cells (LCL1) with puromycin resulted in stable cell lines expressing specific si-RNA against *EBNA3C* (LCL1_sh-E3C), *cyclin D1* (LCL1_sh-CyD1) and sh-RNA sequence that lacks any complementary sequences in the human genome (LCL1_sh-Cont). The selected cells with GFP fluorescence were monitored by fluorescent microscopy. (B) Western blots showing the expression levels of EBNA3C, pRb, Cyclin D1, Cyclin D2 and Cyclin D3 in LCLs. GAPDH was used as the loading control. (C) Approximately 1 million cells were plated into each well of the 6-well plates and cultured at 37°C in complete medium without puromycin. Viable cells from each well were counted by trypan blue exclusion method daily for twenty days using an automated cell counter. The results shown are representative of two independent experiments. Error bars show standard deviations.

In the context of the above-described results, we hypothesized that EBNA3C exploits Cyclin D1/CDK6 to promote LCL proliferation by inactivating pRb. To address this, LCLs were stably transduced with lentiviruses that express short hairpin RNA to silence either EBNA3C (Sh-E3C) or cyclin D1 (Sh-CyD1). The Sh-Control RNA is not complementary to human genome sequences. Stable transduction was verified by GFP expression (Fig. 9A). The expression levels of knocked down genes among these cells were then detected by Western blot analysis (Fig. 9B). The results showed that the level of EBNA3C or Cyclin D1 was knocked down by sh-RNA whereas LCL1 transduced with sh-Control had levels similar to LCL1 (Fig. 9B). The results also showed that pRb expression levels were slightly increased in both EBNA3C and Cyclin D1 knocked down samples, whereas there were no alterations observed for other Cyclin D expression levels (Fig. 9B). In order to determine whether both EBNA3C and Cyclin D1 are critical to maintain the proliferation of EBV transformed cells, a proliferation analysis was done (Fig. 9C). The results showed that the proliferation rate of both wild-type LCL1 and LCL1 infected with the lentivirus control sh-RNA (Sh-Control) expressing physiological level of both EBNA3C and Cyclin D1 was significantly higher than that of LCLs with Sh-E3C and Sh-CyD1 (Fig. 9C). In agreement with the previously published results [20], [49], we also observed that the proliferation rate of LCLs containing Sh-E3C with reduced levels of EBNA3C expression was approximately 3 fold slower than that of control cell-lines (Fig. 9C). Interestingly, the proliferation rate of LCLs with Sh-CyD1 was 50% higher than LCLs with Sh-E3C and only about 1.5 fold lower than that of control. This suggests that other D-type cyclins might be involved in LCL growth, particularly Cyclin D2 which was shown earlier to be associated in EBV mediated lymphomagenesis and probably transcriptionally up-regulated by one of the other major EBV latent antigen LMP1 [45]. However, it is clear from repeated analyses that cyclin D1 knock-down correlates with an increase in doubling time. The results support the notion that EBNA3C and cyclin D1 are critical for driving the growth of EBV transformed cells.

**Figure 10:**
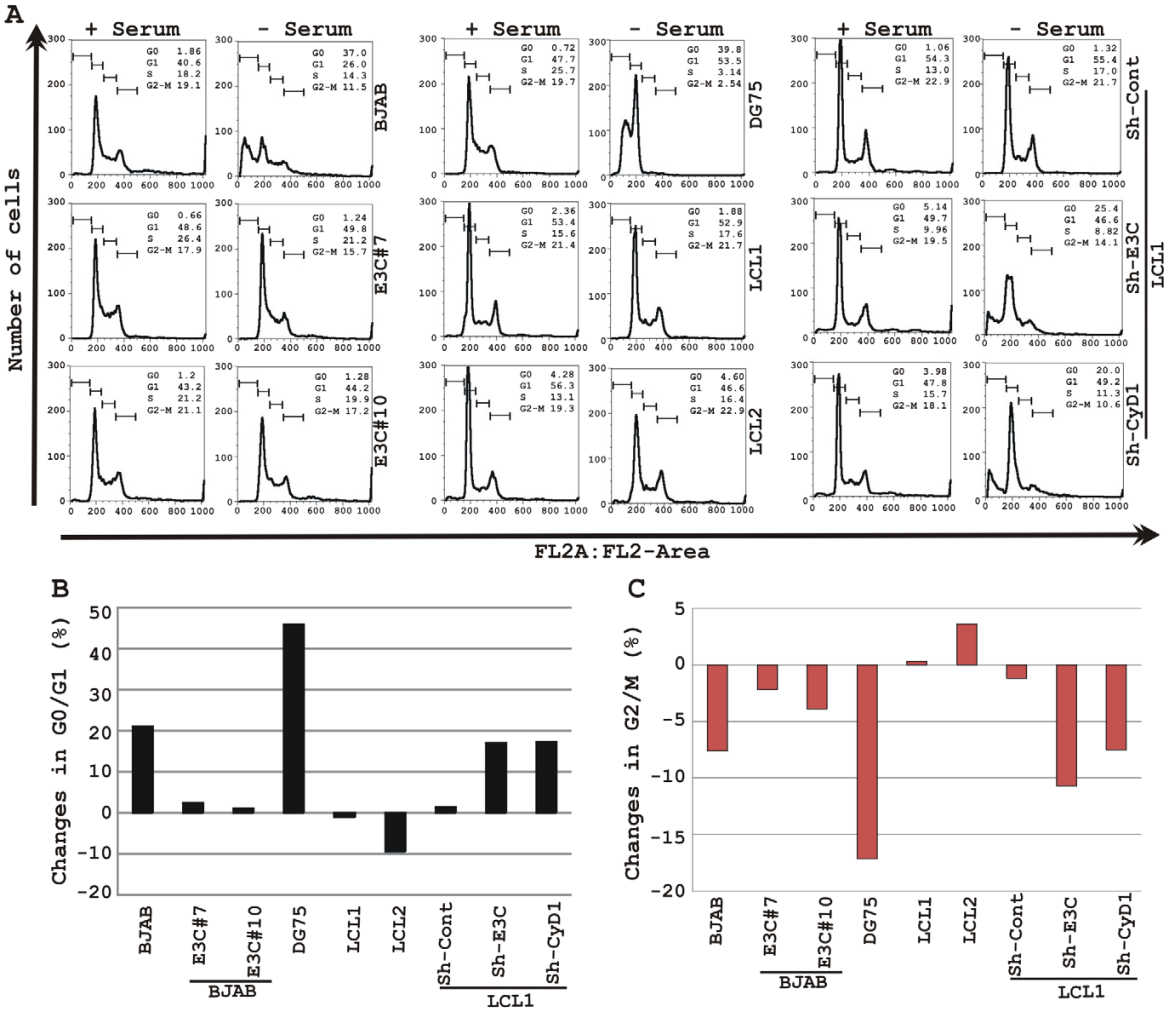
EBNA3C and Cyclin D1 are critical for G1 to S phase progression. A) Cells were grown for 12 h in RPMI medium containing 10% FBS (+ serum) or 0.1% FBS (- serum). Propidium iodide stained cells were analyzed by flow cytometry. The bar diagram represents the change in cell-cycle profile either in (B) G0-G1 phase or (C) G2-M phase due to serum starvation of cells. The results shown are representative of two independent experiments.

It has been shown earlier that both EBV positive cells and cells stably expressing EBNA3C can bypass G1/S phase checkpoint caused by serum starvation [20], [35]. Cell-cycle profiles of cells cultured in medium with 0.1% FBS were analyzed by flow cytometry (Fig. 10). Analyses of serum-starved, EBV negative cell lines BJAB and DG75 and LCLs sh-E3C and sh-CyD1 revealed an increased percentage of cells at the G0/G1 phase of the cell cycle (Fig. 10A, B) and decreased percentage of cells in the G2/M phases (Fig.10A, C). Fig. 10B and 10C represents the difference in both G0/G1 and G2/M phases due to serum starvation, respectively. However, under the same culture conditions, the EBV-positive LCLs - LCL1, LCL2, LCL1-with Sh-control and BJAB-cells stably expressing EBNA3C (E3C# 7 and E3C# 10) continued through the cell-cycle without being arrested at any particular phase (Fig. 10A histograms, B and C). Furthermore, the results also indicated that upon knockdown of both EBNA3C and Cyclin D1, LCLs underwent a substantial degree of apoptosis (Sub G0) in response to serum starvation, similar to EBV negative cell lines, BJAB and DG75 (Fig. 10A). However, there was no sign of apoptosis observed either in BJAB cells stably expressing EBNA3C or wild-type LCLs (Fig. 10A). Altogether, this experiment demonstrated that EBNA3C and Cyclin D1 positively contribute to cell growth in EBV transformed cells and are critical for overriding the G1 block as a result of serum starvation.

## Discussion

The *cyclin D1* gene amplification has been observed in cancers of the breast, head and neck or larynx [50], [51], [52]. Chromosomal rearrangement is another cause of Cyclin D1 over-expression associated with centrocytic lymphomas [53], parathyroid adenomas [54] and mantle cell lymphoma [28]. The obvious association of Cyclin D1 with cancer has led the investigators to uncover its oncogenic properties. In fact, Cyclin D1 was shown to cooperate with the Ras oncoprotein for cell transformation [55].

Earlier reports have suggested that immortalization of primary B-lymphocytes by EBV is accompanied by transcriptional activation of the *cyclin D2* gene but not *cyclin D1* [43], [44], [45]. However, a number of studies showed noticeable changes in Cyclin D1 protein levels in both EBV positive LCLs [35] and EBV positive SCID mice lymphomas [37]. Despite the controversy regarding the Cyclin D1 expression in EBV positive B-lymphoma cells, it is clear that in order to deregulate the entire mammalian cell-cycle, EBNA3C manipulates G1 restriction point through disruption of Cyclin/CDK-pRb-E2F pathway [20].

Cyclin D1 is over-expressed in a variety of human cancers that do not exhibit *cyclin D1* gene amplification or structural abnormalities of the *cyclin D1* locus, which suggests that increased Cyclin D1 stability is a potential mechanism. Mutations of *cyclin D1* at T286 and P287 have been found in human tumors [24] and alter Cyclin D1 nuclear localization as well as stability. Our data showed that both EBV infection in primary B-cells and EBV persistence in cancer cell lines increased protein stability. However the *cyclin D1* mRNA level was unchanged. Similar to virus infection, EBNA3C gene expression increased Cyclin D1 levels without altering mRNA levels. It is important to determine if these effects also occur *in vivo*.

The results presented here also demonstrated that the expression of Cyclin D2 and D3 were up-regulated in quiescent cells infected with EBV probably through distinctly different mechanisms. EBV infection or its transforming protein latent membrane protein 1 (LMP1) up-regulates Cyclin D2 expression in primary B-lymphocytes and Burkitt's lymphoma cells [45]. None of the studies have shown an important role for Cyclin D3 in EBV-mediated cell transformation. Studies have suggested that the D-type cyclins may have non-overlapping functions at specific steps in B-cell differentiation [32], and that the expression of different D-type cyclins may be influenced by EBV infection through distinctive pathways. Thus, a potential mechanism which involves the contribution of Cyclin D1 in EBV-mediated B-cell transformation is the constitutive induction of these key cell-cycle regulators which leads to pRb hyper-phosphorylation and uncontrolled cell proliferation.

Several lines of evidence suggest that Cyclin D1 is targeted by the E3 ligase, SCF^FBX4-αB crystallin^ for degradation [26]. Elevated expression of FBX4 and αB crystallin is also found to trigger the destruction of wild-type Cyclin D1, but not the phosphorylation-deficient Cyclin D1 mutant, D1T286A [26]. Thus, impairment of SCF^FBX4-αB crystallin^ function may also account for Cyclin D1 overexpression. Data from the ubiquitination assay showed that EBNA3C efficiently inhibits Cyclin D1 poly-ubiquitination, which led us to speculate that EBNA3C may interact with this particular E3 ligase and inhibit its ability to ubiquitinate Cyclin D1. The SCF^Skp2^ ligase has also been shown to be involved in the degradation of Cyclin D1 [56], [57], [58], and knockdown of Skp2 molecule promoted marked accumulation of Cyclin D1 [57]. EBNA3C interacts with SCF components to regulate the stability of p27^KIP1^ and pRb [12], [17]. It is likely EBNA3C inhibition of SCF^Skp2^ reduces Cyclin D1 ubiquitination. EBNA3C may be a deubiquitinase or associate with one to regulate the stability of Mdm2 [18] and likely Cyclin D1. Since the expression level of Cyclin D1 is related to the potential for malignancy and the prognosis of a variety of cancers [30], [31], revealing the mechanisms governing the ubiquitin-proteasome mediated degradation of Cyclin D1 is of importance in designing therapeutic interventions. Conceivably, this approach could amplify the therapeutic window using Cyclin D1 as a target and enhance the efficacy of conventional drugs against EBV mediated oncogenesis.

We have shown earlier that EBNA3C can interact with Cyclin D1 using an *in vitro* GST-pulldown experiment [16]. Here, we examined the molecular association between EBNA3C and Cyclin D1 complexes to obtain a more in-depth understanding of the different domains of EBNA3C that modulate the activity of Cyclin D1 which will lead to further understanding the basic mechanism by which EBV regulates the mammalian cell-cycle. EBNA3C associates with Cyclin D1 via the same N-terminal domain, residues 130-190, that has been shown to bind many critical cell-cycle regulators [18] including other Cyclins - A and E [16]. In addition, a recent genetic study using recombinant EBV expressing conditionally active EBNA3C showed that deletion of this particular domain could not support cell proliferation of EBV transformed LCLs, signifying the importance of this domain within EBNA3C [49]. The association of EBNA3C with different Cyclins suggests is perhaps cell-cycle dependent and one may replace another depending on the stage in the cell-cycle, which ultimately leads to aberrant cell proliferation in EBV transformed cells. The previously published data and the data herein were generated using asynchronously growing cells; therefore it would be interesting to further elucidate these interactions in a cell-cycle dependent manner. However, using chemical synchronization is likely to distort the true activities underlying EBNA3C function with Cyclin complexes. Nevertheless, we will be undertaking this line of experimentation in the near future.

To promote G1-S phase transition, nuclear localization of Cyclin D1 is critical and it occurs either via decreased proteolysis in cytoplasm which facilitates nuclear import or through inhibition of GSK-3β function which triggers nuclear export via phosphorylation at T286 [27], [59]. Immunofluorescent studies showed that EBNA3C expression enforces nuclear localization of Cyclin D1. Increased stability and nuclear accumulation of Cyclin D1 in the presence of EBNA3C prompted us to examine whether EBNA3C can also negatively regulate GSK-3β function linked to the stability of Cyclin D1. Indeed, our data show that EBNA3C forms a complex with GSK-3β and significantly reduces its kinase activity toward Cyclin D1, thereby enhancing the nuclear retention of Cyclin D1. Altogether, these data suggest that either by increasing nuclear import by blocking the poly-ubiquitination level of Cyclin D1 or inhibiting nuclear export of Cyclin D1 via inhibiting the kinase activity of its negative regulator GSK-3β, EBNA3C would likely ensure the efficient nuclear accumulation of Cyclin D1 during G1-phase. However, we cannot eliminate the possibility that EBNA3C may also facilitate Cyclin D1 nuclear accumulation through additional strategies.

Cyclin D1 is central to the coordination of the cell-cycle progression at the G1 to S phase transition by integrating the control of pRb phosphorylation with the transcriptional activity of E2F [60]. Cyclin D1 in association with its binding partner, CDK4 or 6 phosphorylates pRb to facilitate S phase entry [60]. Previously we have shown that EBNA3C enhances the kinase activity of Cyclin A/CDK2 complex [15] and recruits an E3 ligase SCF^Skp2^ to degrade pRb [17]. Similarly, here we show that by an *in vitro* kinase assay EBNA3C increases the activity of Cyclin D1/CDK6 complex toward both Histone H1 and a truncated mutant of pRb. Moreover, EBNA3C in conjunction with Cyclin D1/CDK6 complex increases pRb poly-ubiquitination and thereby enhances its degradation process. In addition, we show EBNA3C coupled with Cyclin D1/CDK6 complex significantly abolishes the growth suppressive function of pRb in Saos-2 cells.

Studies using serum starved conditions have shown that both EBV and its essential nuclear antigen, EBNA3C can bypass G1 restriction point probably through disruption of Cyclin/CDK-pRb-E2F pathway [21], [36]. LMP1 has also been shown to be associated with resistance to G1 arrest during serum starvation [36]. Taking advantage of these findings, together with the use of sh-RNA mediated gene knockdown strategies, we have generated knockdown lymphoblastoid cell-lines targeting both *EBNA3C* and *cyclin D1* transcripts and assayed for cellular proliferation to carefully determine the plausible role of both of these viral and cellular oncoproteins. Indeed, our results show that both EBNA3C and Cyclin D1 are critical for efficient proliferation of EBV infected B-cells. Moreover, the results point out that upon knockdown of these gene products, cells undergo significant apoptosis, probably through induction of the activities of the tumor suppressors – p53 and pRb. Earlier results [14] and the data herein adequately show that EBNA3C critically regulates the growth suppressive properties of both p53 and pRb.

**Figure 11:**
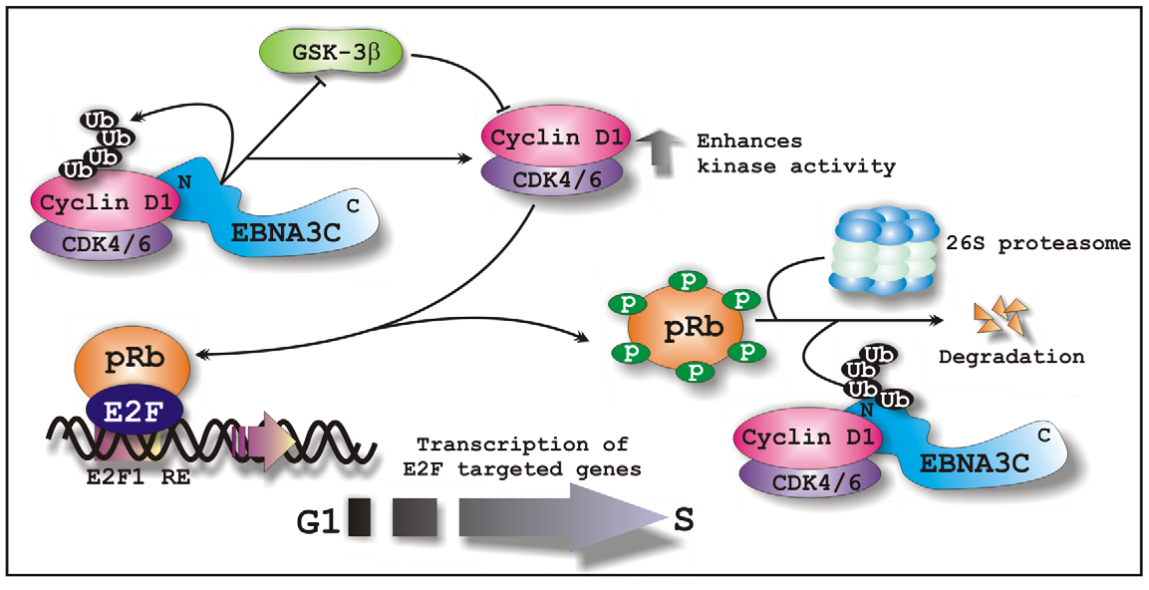
A schematic illustration of how EBNA3C regulates Cyclin D1 stability and functions to facilitate G1 to S phase transition in EBV positive cells. In EBV transformed cells, EBNA3C forms a complex with Cyclin D1 and augments its stability through inhibiting poly-ubiquitination and blocking GSK3β activity. EBNA3C further enhances the kinase activity of Cyclin D1/CDK6 complex and recruits its activity to facilitate the ubiquitination and subsequent degradation of hyperphosphorylated form of pRb, which in turn releases E2F transcription factor from an inhibitory constraint and enables the expression of genes required for entry into the S phase.

Overall, we have shown in this report that the essential EBV latent antigen, EBNA3C physically interacts with and stabilizes Cyclin D1 by blocking nuclear export or inhibiting the poly-ubiquitination. In addition, EBNA3C alters pRb phosphorylation as well as stability by enhancing Cyclin D1/CDK6 kinase activity, thereby nullifying pRb mediated growth suppressive activity (Fig. 11). Furthermore, knockdown of both EBNA3C and Cyclin D1 expression by lentivirus-delivered sh-RNA demonstrated that both EBNA3C and Cyclin D1 play a critical role in cell proliferation in EBV transformed cells. Thus, the present study provides an insight into the mechanisms linked to the development of EBV-associated B-cell lymphomas through the enhancement of a major cell-cycle component, Cyclin D1, which is known to orchestrate the activities of a vast range of cellular networks that are important in the development of human cancers.
